# Combining gene expression, demographic and clinical data in modeling disease: a case study of bipolar disorder and schizophrenia

**DOI:** 10.1186/1471-2164-9-531

**Published:** 2008-11-07

**Authors:** Jan Struyf, Seth Dobrin, David Page

**Affiliations:** 1 Department of Computer Science, Katholieke Universiteit Leuven, Celestijnenlaan 200A, 3001 Leuven, Belgium; 2 Marshfield Clinic Research Foundation, Marshfield, WI 54449, USA; 3 Monsanto Company, High-Throughput Genetics, 3302 SE, Convenience Boulevard, Ankeny, IA 50021, USA; 4 Department of Biostatistics and Medical Informatics and Department of Computer Sciences, University of Wisconsin, Medical Science Center, 1300 University Avenue, Madison, WI 53706, USA

## Abstract

**Background:**

This paper presents a retrospective statistical study on the newly-released data set by the Stanley Neuropathology Consortium on gene expression in bipolar disorder and schizophrenia. This data set contains gene expression data as well as limited demographic and clinical data for each subject. Previous studies using statistical classification or machine learning algorithms have focused on gene expression data only. The present paper investigates if such techniques can benefit from including demographic and clinical data.

**Results:**

We compare six classification algorithms: support vector machines (SVMs), nearest shrunken centroids, decision trees, ensemble of voters, naïve Bayes, and nearest neighbor. SVMs outperform the other algorithms. Using expression data only, they yield an area under the ROC curve of 0.92 for bipolar disorder versus control, and 0.91 for schizophrenia versus control. By including demographic and clinical data, classification performance improves to 0.97 and 0.94 respectively.

**Conclusion:**

This paper demonstrates that SVMs can distinguish bipolar disorder and schizophrenia from normal control at a very high rate. Moreover, it shows that classification performance improves by including demographic and clinical data. We also found that some variables in this data set, such as alcohol and drug use, are strongly associated to the diseases. These variables may affect gene expression and make it more difficult to identify genes that are directly associated to the diseases. Stratification can correct for such variables, but we show that this reduces the power of the statistical methods.

## Background

The Stanley Neuropathology Consortium [1] recently made a large (over 300 sample) data set publicly available on gene expression in the brains of deceased individuals with bipolar disorder or schizophrenia, as well as controls. In addition the data contains limited demographic and clinical history information, including gender and history of smoking, alcohol and drug use. This paper presents a retrospective statistical study on this data set, in which we address the following three questions:

Q1. Can either bipolar disorder or schizophrenia be distinguished from control purely on the basis of gene expression profile?

Q2. Does addition of the demographic and clinical history data further improve the ability to distinguish bipolar disorder or schizophrenia from control?

Q3. Is there a significant difference between the abilities of different widely-used data analysis algorithms to make these distinctions?

We show that bipolar disorder and schizophrenia each can be distinguished from control, based on gene expression alone, significantly better than chance – in fact with areas under the Receiver Operating Characteristic (ROC) curve (AUC) of 0.91 (schizophrenia vs. control) and 0.92 (bipolar disorder vs. control). While area under the ROC curve indicates how well one can distinguish across a range of specificities (with 0.5 being no better than chance and 1.0 being perfect distinction), it is also worth noting that for each task, a sensitivity of 0.85 can be achieved when operating at a specificity of 0.9. Moreover, by taking demographic information and clinical history into account (see Table 1), performance improves to an AUC of 0.94 for schizophrenia vs. control and to an AUC of 0.97 for bipolar disorder vs. control. To our knowledge, this is the first statistical comparison of the efficacy of using a combination of gene expression data and clinical history data against using gene expression data alone. With regard to question Q3, the paper shows that support vector machines (SVMs) significantly outperform the other most widely used algorithms for statistical classification and machine learning for these tasks.

**Table 1:** Demographic and clinical features

Feature	Value (encoding)	Control	Schiz.	Bipolar
Age		44 ± 8	43 ± 9	45 ± 10

Sex	Male (1)	81	86	53
	Female (-1)	31	29	52

PMI		29 ± 13	31 ± 15	38 ± 17

Brain pH		6.6 ± 0.3	6.4 ± 0.3	6.4 ± 0.3

Left brain	Frozen (1)	51	57	59
	Fixed (-1)	61	58	46

Brain region	FrontalBA46 (1)	101	104	94
	FrontalBA46/10 (-1)	11	11	11

HSV 1 OD Z-score		0.1 ± 1.0	-0.2 ± 0.9	-0.0 ± 0.8

HSV 2 OD Z-score		-0.2 ± 0.5	-0.1 ± 0.7	0.3 ± 1.3

Smoking at TOD	Yes (1)	29	71	47
	No (-1)	29	19	18
	Unknown (0)	54	25	40

Alcohol use	Unknown (1)	0	0	4
	Little or none (2)	56	35	12
	Social (3)	38	22	24
	Moderate in past (4)	4	10	16
	Moderate in present (5)	8	10	10
	Heavy in past (6)	6	11	16
	Heavy in present (7)	0	27	23

Drug use	Unknown (1)	0	6	0
	Little or none (2)	97	52	32
	Social (3)	7	7	8
	Moderate in past (4)	5	13	21
	Moderate in present (5)	3	8	12
	Heavy in past (6)	0	11	6
	Heavy in present (7)	0	18	26

Rate of death	Sudden (1)	110	91	96
	Possible anoxia (2)	0	18	6
	Slow death (3)	2	3	0
	Mechanical ventilator (4)	0	3	3

This table lists the distribution (count or mean ± standard deviation) of the demographic features in the three classes (control, schizophrenia, bipolar disorder). Because most classification techniques are restricted to numerical features only, we reencode each nominal feature as a numeric feature. The numerical encoding is listed between parentheses after each nominal feature value.

Furthermore, we found that some variables in this data set, such as alcohol and drug use, are strongly associated to the diseases. Given that these variables may affect gene expression, they may make it more difficult to identify genes that are directly associated to the diseases. (We discuss this point in detail later in the text.) We have investigated if post-stratification can correct for such variables, but we found that it significantly reduces the predictive accuracy of the statistical methods.

### Data

The expression data set was obtained from the Stanley Neuropathology Consortium [1]. The records utilized in this study are a subset of the entire collection of data. The data set contains 115 schizophrenia patients, 105 patients with bipolar disorder and 112 controls. For each subject, it includes annotated gene expression data and demographic and clinical information. All data was analyzed un-blinded. Diagnosis and criteria have been described previously by Torrey and colleagues [2]. As described in the same report by Torrey and colleagues, the recreational or prescription status of drugs for each of the donors was largely unknown, because the researchers relied on post-mortem urine toxicology screens that are not always conducted.

The expression data was obtained using Affymetrix Human Genome U133A GeneChip oligonucleotide arrays containing 22,283 probe sets (Affymetrix, Santa Clara, CA). Probe level data was summarized using the GC content adjusted robust multi-array average (RMA) method [3]. The data set includes the GC-RMA value of each probe set as a numerical feature.

The data set records, besides expression data, also demographic and clinical information about the subjects. Table 1 lists the recorded demographic and clinical features with their distribution in the three classes. For numeric features, it lists the mean and standard deviation, and for nominal features, the class-wise count of each feature value.

### Algorithms

We define two binary classification tasks on the data set: schizophrenia versus control and bipolar versus control. For each task, we compare the following six classification techniques: support vector machines, nearest shrunken centroids, decision trees, ensemble of voters, naïve Bayes, and nearest neighbor. We briefly describe each technique. The section "Methods" lists the software packages that we use and explains how the parameters of the different algorithms are set.

#### Support vector machines

**Figure 1:**
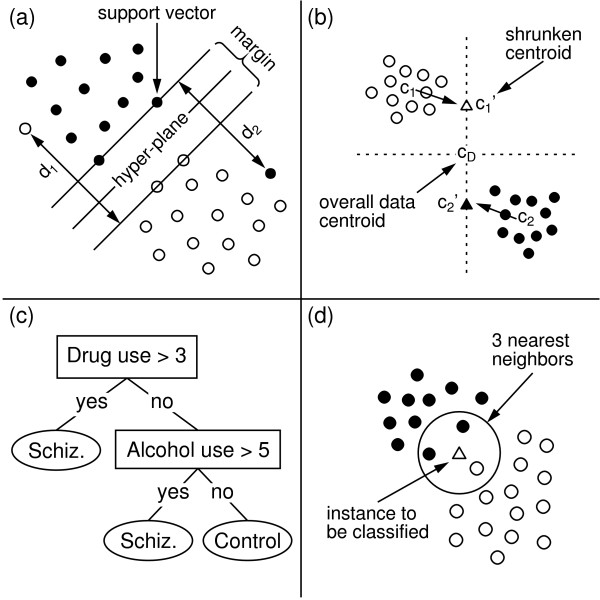
Illustration of the (a) support vector machines, (b) nearest shrunken centroids, (c) decision trees, and (d) nearest neighbor methods.

Support vector machines (SVMs, [4]) belong to the family of generalized linear models. We employ linear SVMs, which exhibit good classification performance on gene expression data [5]. A linear SVM is essentially an (*n-1*)-dimensional hyper-plane that separates the instances of the two classes in the *n*-dimensional feature space. Figure 1a illustrates this for the two dimensional case: the hyper-plane reduces here to a line, which separates the empty (class 1) and filled (class 2) circles. The hyper-plane maximizes the margin with the closest training instances. These instances are called the "support vectors" because they fix the position and orientation of the hyper-plane. Linear SVMs assume that the training data is linearly separable. If this is not the case, then SVMs rely instead on the concept of a soft margin [6]. In the evaluation, we use a soft margin SVM, which minimizes, in addition to the margin, also the sum of the distances to the training instances that are incorrectly classified by the hyper-plane (the *d*_*i *_in Figure 1a).

#### Nearest shrunken centroids

Nearest shrunken centroids (NSC, [7]) is a technique designed for classifying gene expression data. NSC represents each class by its centroid (mean feature vector) and classifies new instances by assigning them the class of the closest centroid. NSC shrinks the class centroids (*c*_*i *_in Figure 1b) in the direction of the overall data centroid. This has the effect that components of a class centroid that after shrinkage are equal to the corresponding components of the overall centroid become irrelevant to the classification process. This occurs for the horizontal component of the class centroids in Figure 1b. As a result, NSC implicitly performs a kind of feature selection.

#### Decision trees

Decision trees (DTs, [8]) are tree-shaped symbolic models with tests on the feature values in the internal nodes, and class labels in the leaves (Figure 1c). DTs classify a new instance by sorting it down the tree, according to the tests in the nodes, until it reaches a leaf; the label of the leaf becomes the predicted class of the new instance. C4.5 [8] is a well-known algorithm for constructing decision trees. C4.5 builds a DT top-down, by recursively partitioning the data at each step by a test comparing a feature to a value. At each node, the algorithm selects the test that maximizes a heuristic function called information gain ratio. The better a test is able to separate the instances of the two classes, the higher its information gain ratio. Then, it partitions the training instances based on the selected test, and finally it recursively repeats the same procedure to construct a sub-tree for each subset in the partition. C4.5 creates a leaf if all remaining instances belong to the same class or if there are fewer instances than a user defined threshold. The label of the leaf is the majority class of the instances it covers. After building the tree, C4.5 prunes back some parts to reduce the expected error on new instances.

#### Ensemble of voters

Ensemble of voters (EOV, [9]) is a simple ensemble method. An EOV model is a set of decision stumps. Decision stumps are decision trees that consist of precisely one test node with two leaves. The EOV model includes one decision stump for each of the top *N *feature value tests ranked by the information gain score [8]. To obtain a prediction for a new instance, the model combines the predictions of the stumps by means of majority voting: the predicted class is the class predicted by more than *N/2 *stumps.

#### Naïve Bayes

Naïve Bayes (NB, [10]) is a statistical classifier based on Bayes rule. Its name comes from the strong (naïve) statistical independence assumptions that it makes. In spite of these strong assumptions, it often works remarkably well in practice. NB predicts a class with the rule: $class=\underset{c\in classes}{\arg\max}P(c)\prod_{i}P(feature_{i}=value_{i}|c)$. It estimates *P(c) *and *P*(*feature*_*i *_= *value*_*i*_|*c*) from the training data. Note that NB assumes nominal features, which means that numerical features must be discretized prior to running NB.

#### Nearest neighbor

*k*-nearest neighbor (*k*NN, [11]) classifies a new instance as the majority class of its *k *closest training instances in the feature space. For example, 3NN in Figure 1d assigns the class "black" to the new instance (indicated with a triangle).

### Evaluation

We evaluate the performance of the different classification techniques by means of Receiver Operating Characteristic (ROC) curves. ROC analysis allows us to simultaneously compare classifiers for different misclassification costs and class distributions [12]. It is based on the notions of "true positive rate" (TP, also known as sensitivity or recall) and "false positive rate" (FP, also known as 1.0 – specificity). Given two classes "positive" and "negative", TP rate is the proportion of correctly predicted positive examples, and FP rate is the proportion of negative examples that are incorrectly predicted positive. The vertical axis of a ROC diagram represents TP rate, and the horizontal axis FP rate. Each classifier corresponds to a point on this diagram. The closer the point is to the upper-left corner (TP rate = 1, FP rate = 0), the better the classifier.

Most classifiers provide confidence scores for their predictions. For such classifiers, a ROC curve can be constructed. We present such a curve for each classifier and report the corresponding "area under curve" (AUC), which is defined as the area between the ROC curve and the horizontal axis. To obtain a measure for the predictive performance of the models, we estimate the ROC curves using a 10-fold cross validation procedure. Details about this evaluation procedure can be found in the section "Methods". We use a *t*-test to assess if the AUC difference between two classifiers is significant and report the corresponding *p*-value.

## Results and discussion

### Classifier performance

We compare the six classification techniques in the context of two classification tasks: schizophrenia versus control and bipolar versus control. In a first set of experiments, the data consist of only the gene expression features (Figures 2, 3, 4, 5, 6, 7), and in a second batch, the data include both demographic and clinical features as well as gene expression (Figures 8, 9, 10, 11, 12, 13).

**Figure 2:**
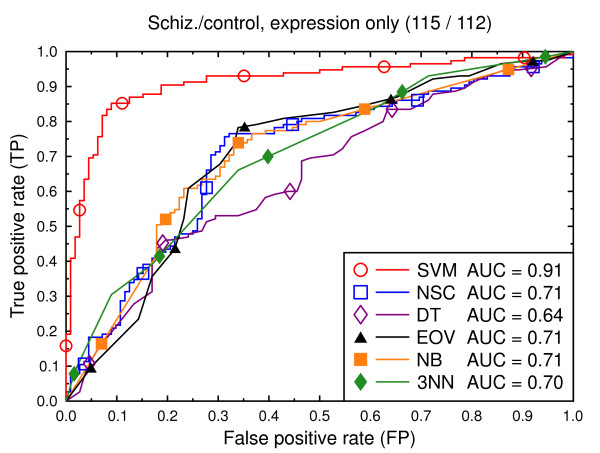
ROC curves, schizophrenia/control, expression data.

**Figure 3:**
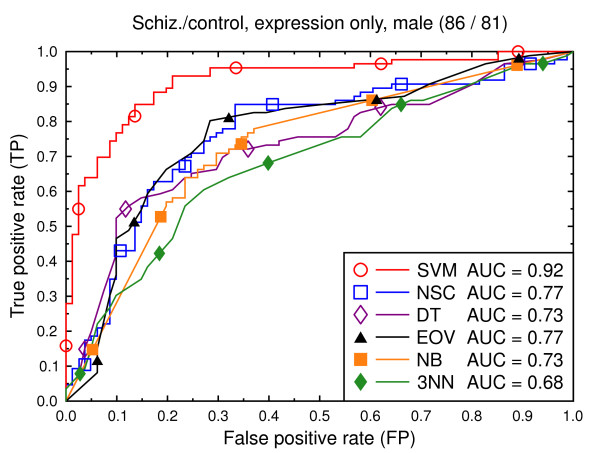
ROC curves, schizophrenia/control, expression data, male subjects.

**Figure 4:**
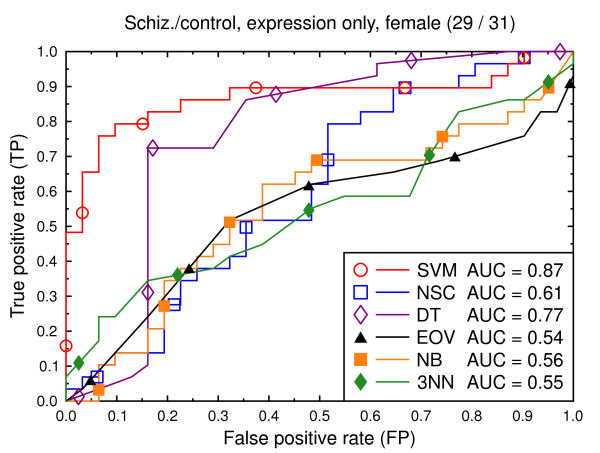
ROC curves, schizophrenia/control, expression data, female subjects.

**Figure 5:**
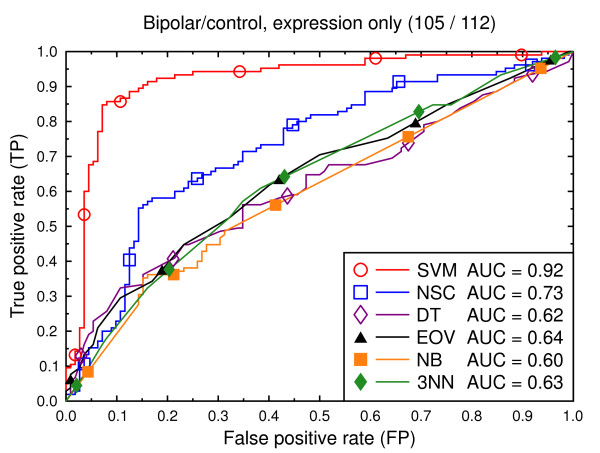
ROC curves, bipolar/control, expression data.

**Figure 6:**
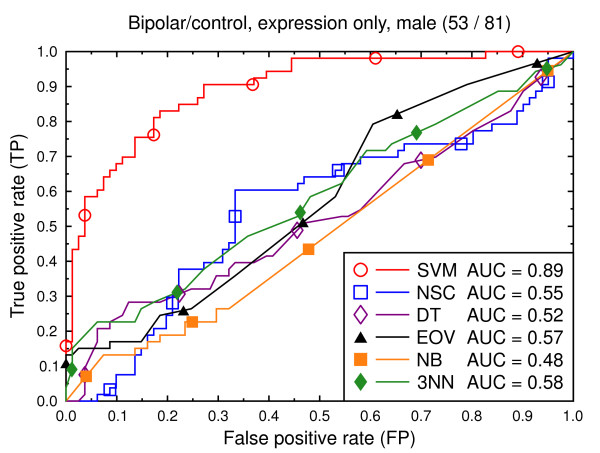
ROC curves, bipolar/control, expression data, male subjects.

**Figure 7:**
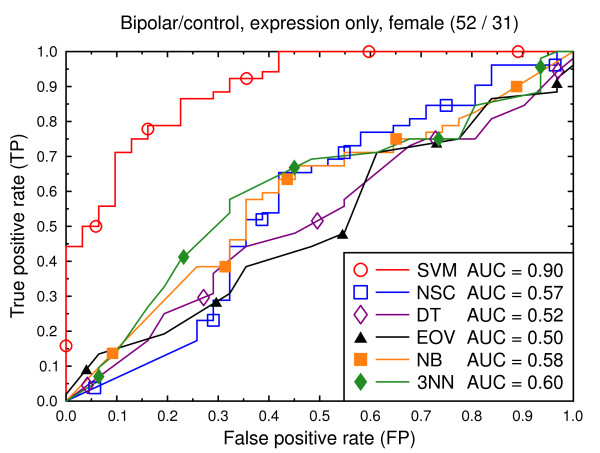
ROC curves, bipolar/control, expression data, female subjects.

**Figure 8:**
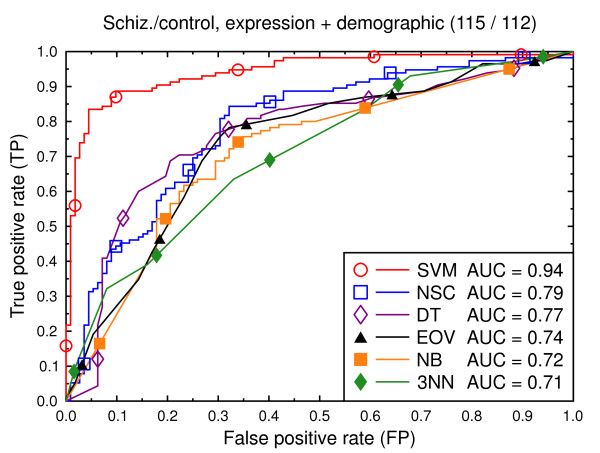
ROC curves, schizophrenia/control, demographic, clinical, and expression data.

**Figure 9:**
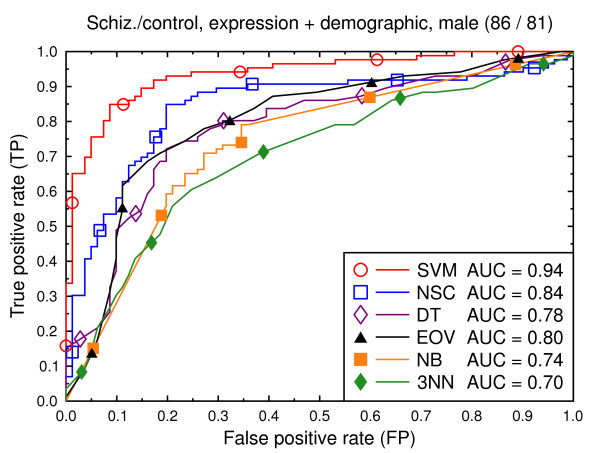
ROC curves, schizophrenia/control, all data, male subjects.

**Figure 10:**
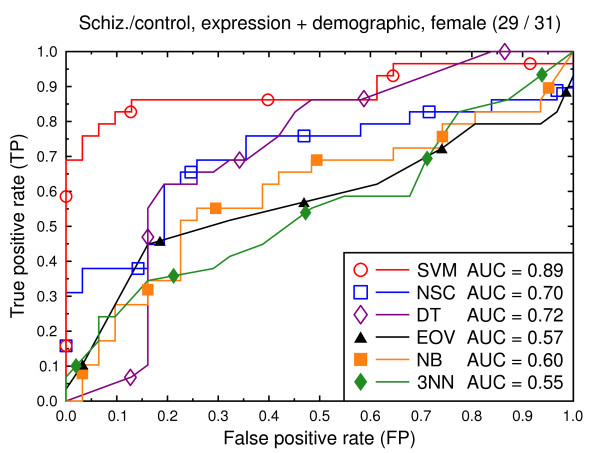
ROC curves, schizophrenia/control, all data, female subjects.

**Figure 11:**
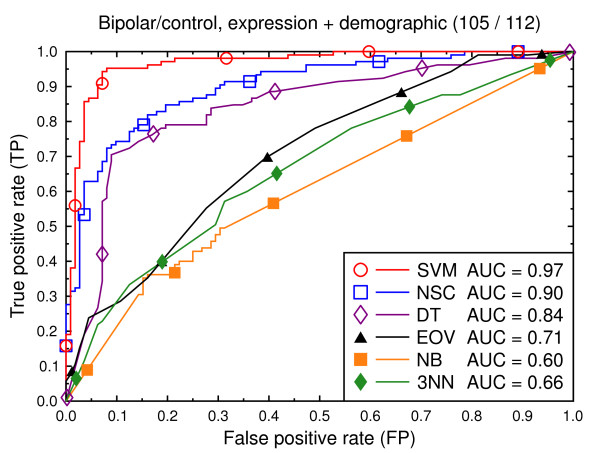
ROC curves, bipolar/control, demographic, clinical, and expression data.

**Figure 12:**
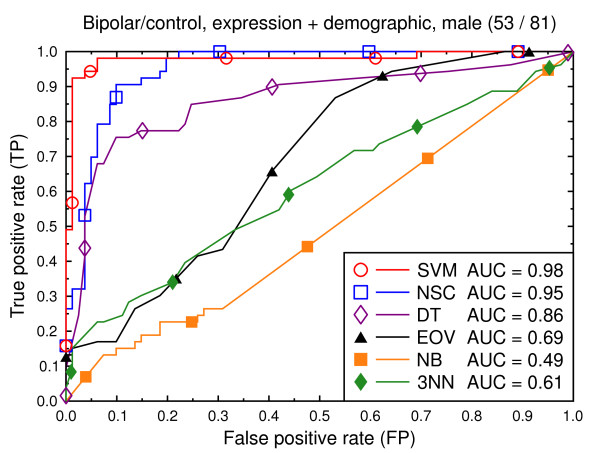
ROC curves, bipolar/control, all data, male subjects.

**Figure 13:**
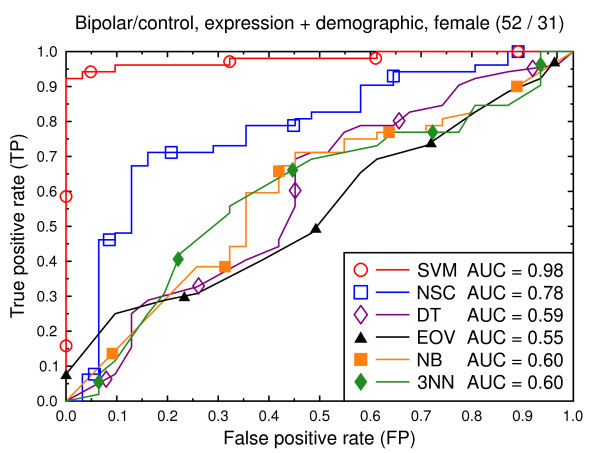
ROC curves, bipolar/control, all data, female subjects.

Figure 2 compares the classification techniques for the schizophrenia versus control task, using only the gene expression data. SVM outperforms the other techniques. It yields a cross validated AUC of 0.91, which is significantly better than NSC (AUC = 0.71, *p *= 0.002), DT (AUC = 0.64, *p *= 0.0001), EOV (AUC = 0.71, *p *= 0.0001), NB (AUC = 0.71, *p *= 0.0004), and 3NN (AUC = 0.70, *p *= 0.0002). The same holds for the bipolar versus control task (Figure 5). SVM (AUC = 0.92) outperforms the other techniques. The second best technique is NSC (AUC = 0.73, *p *= 0.01).

We also present experiments on data for male subjects only (Figure 3 and Figure 6) and for female subjects only (Figure 4 and Figure 7), to assess if the diseases can be better predicted if data of only one sex is used. The SVM result AUC = 0.91 on the combined data of Figure 2 for schizophrenia versus control is, however, not significantly different from the result for male subjects (Figure 3, AUC = 0.92, *p *= 0.9) or that for female subjects (Figure 4, AUC = 0.87, *p *= 0.4). The same holds for the bipolar versus control task. We hypothesize that this is because the data sets with subjects of one sex only are much smaller than the combined data, so that there is less training data for each model. Even if classification is easier for such data, this is offset by the smaller data size.

Figures 8, 9, 10, 11, 12, 13 present a similar set of experiments for the data set that includes demographic and clinical data in addition to the gene expression data. Adding demographic and clinical information improves classification performance. SVM, for example, performs better on the schizophrenia versus control task with demographic information (Figure 8, AUC = 0.94) than without such additional information (Figure 2, AUC = 0.91, *p *= 0.06). The same holds for the other classification techniques and for the bipolar versus control task (Figure 11 versus Figure 5).

We again present experiments for male and female subjects separately (Figures 9, 10, 12 and 13), this time with the demographic and clinical data included. The conclusion from this set of experiments is similar to the previous conclusion: separating the subjects by sex does not significantly improve the classification performance for SVMs.

The superior performance of SVMs when compared to the other classification algorithms can be understood based on the properties of gene expression data. Gene expression data is typically characterized by a high dimension combined with a relatively low number of samples. For example, the present data set records the expression level of 22,283 probe sets for a number of samples that is two orders of magnitude smaller. Many classification algorithms are known to perform poorly on such high dimensional data. SVMs, on the other hand, are well suited to this setting because their classification performance can be independent of the dimensionality of the feature set [13]: their performance rather depends on the margin with which they separate the samples (Figure 1a). This explains the good performance of SVMs on high dimensional data. Additional empirical evidence is that SVMs are known to perform well on text classification problems (where each word in the vocabulary represents a dimension) [13]. Previous studies on gene expression data also illustrate the good performance of SVMs [5,9].

Note that the above discussion does not imply that SVMs will always outperform other algorithms on gene expression data. For example, NSC, which implicitly performs dimensionality reduction (recall that it shrinks the class centroids towards the overall data centroid), has also been shown to work well on gene expression data [7,9]. Therefore, it is common practice in machine learning to evaluate different classification algorithms on a new data set and based on this evaluation select the one that works best. This is also the approach that we follow in this work.

### Most relevant features

To asses which features are most relevant to each of the classification tasks, we apply two techniques: (a) ranking the features by their *p*-value, and (b) ranking the features by their SVM weight. The first technique performs, for each feature, a two-sided *t*-test comparing the feature's values in the two classes. It then ranks the features by their *t*-test's *p*-value. Besides the *p*-values, we also report *q*-values [14]. *q*-values measure significance in terms of the false discovery rate. For example, if all features with a *q*-value ≤ 5% are called significant, then 5% of these features may be false discoveries, that is, their mean value in the two classes may be actually identical. We use the software QVALUE developed by Storey [15] to compute the *q*-values. The second technique ranks the features by the weight that the SVM classifier assigns to each feature in the linear equation of its classification hyper-plane. The larger the absolute value of the SVM weight, the more important the feature is to the classification task.

**Figure 14:**
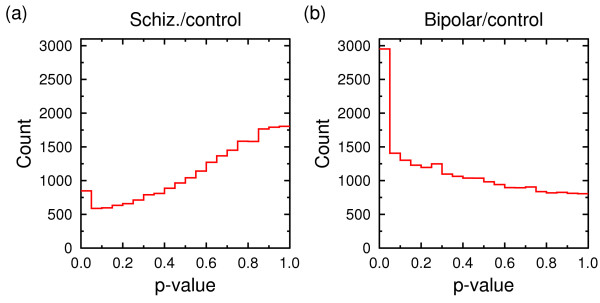
***p*-value histogram for (a) schizophrenia/control and (b) bipolar/control (expression data, all subjects)**. *p*-values of truly null features are distributed uniformly, while *p*-values of significant features are clustered around 0.0. This translates to a flat histogram with a peak at 0.0, as in (b). The *p*-values in (a) are biased towards 1.0 causing the *q*-value estimates to be conservative. The reason for observing such biased distributions is currently not very well understood [59].

The QVALUE software computes, in addition to the *q*-values, also an estimate of the proportion π_0 _of truly null features. For each of the schizophrenia versus control tasks it estimates π_0 _to be 1.0, that is, no significant features; for the bipolar versus control tasks, π_0 _ranges from 0.54 to 0.72. Note that the estimate for schizophrenia versus control is conservative (an overestimate). QVALUE makes certain assumptions about the *p*-value distribution of the data, which do not hold in this case (cf. Figure 14). It is interesting that, even though QVALUE estimates that there are no significant individual features, it is still possible to build classification models that are highly accurate on previously unseen data. (Recall that SVM yields a cross-validated AUC of 0.91 for schizophrenia versus control.) This is partly because the QVALUE estimate is conservative. But it also is partly because classification techniques do not rely on a single feature, but exploit the combined effect of the set of most relevant features. Therefore, obtaining an accurate classifier is possible even if there are no individual significant features.

Table 2 (schizophrenia versus control) and Table 3 (bipolar versus control) rank the features by *p*-value. The left panel of each table shows results based on expression data only; the right panel presents results that include the demographic and clinical features as well. Each table consists of three parts: the top part contains the rankings for all subjects combined, the middle part the male subjects' rankings, and the bottom part the female subjects' rankings.(see additional file 3)

**Table 2:** Genes sorted by *p*-value, schizophrenia versus control

Expression data only					Demographic, clinical, and expression data				
	
All subjects					All subjects				
	
*p*-value	*q*-value	ID	GenBank	Symbol	*p*-value	*q*-value	ID	GenBank	Symbol
	
3.92E-08	8.74E-04	221011_s_at	NM_030915	LBH	1.39E-10	3.11E-06	Drug use		
1.02E-07	1.13E-03	204326_x_at	NM_002450	MT1X	2.88E-09	3.21E-05	Alcohol use		
2.47E-07	1.84E-03	208581_x_at	NM_005952	MT1X	3.92E-08	2.92E-04	221011_s_at	NM_030915	LBH
6.37E-07	3.55E-03	202688_at	NM_003810	TNFSF10	1.02E-07	5.67E-04	204326_x_at	NM_002450	MT1X
1.73E-06	6.20E-03	209735_at	AF098951	ABCG2	2.47E-07	1.10E-03	208581_x_at	NM_005952	MT1X
1.91E-06	6.20E-03	212859_x_at	BF217861	MT1E	6.37E-07	2.37E-03	202688_at	NM_003810	TNFSF10
1.95E-06	6.20E-03	205208_at	NM_012190	ALDH1L1	1.73E-06	4.83E-03	209735_at	AF098951	ABCG2
2.77E-06	7.73E-03	209959_at	U12767	NR4A3	1.91E-06	4.83E-03	212859_x_at	BF217861	MT1E
3.25E-06	8.04E-03	213921_at	NM_001048	SST	1.95E-06	4.83E-03	205208_at	NM_012190	ALDH1L1
6.39E-06	1.37E-02	207547_s_at	NM_007177	FAM107A	2.77E-06	6.19E-03	209959_at	U12767	NR4A3
6.96E-06	1.37E-02	205984_at	NM_001882	CRHBP	3.25E-06	6.59E-03	213921_at	NM_001048	SST
7.36E-06	1.37E-02	221950_at	AI478455	EMX2	6.39E-06	1.13E-02	207547_s_at	NM_007177	FAM107A
9.28E-06	1.59E-02	206001_at	NM_000905	NPY	6.96E-06	1.13E-02	205984_at	NM_001882	CRHBP
1.02E-05	1.63E-02	212185_x_at	NM_005953	MT2A	7.36E-06	1.13E-02	221950_at	AI478455	EMX2
1.61E-05	2.39E-02	209047_at	AL518391	AQP1	7.62E-06	1.13E-02	Smoking at TOD		
1.75E-05	2.44E-02	206461_x_at	NM_005951	MT1H/P2	9.28E-06	1.29E-02	206001_at	NM_000905	NPY
2.06E-05	2.69E-02	202936_s_at	NM_000346	SOX9	1.02E-05	1.34E-02	212185_x_at	NM_005953	MT2A
2.96E-05	3.50E-02	202917_s_at	NM_002964	S100A8	1.61E-05	1.99E-02	209047_at	AL518391	AQP1
2.98E-05	3.50E-02	213791_at	NM_006211	PENK	1.75E-05	2.06E-02	206461_x_at	NM_005951	MT1H/P2
3.43E-05	3.82E-02	205630_at	NM_000756	CRH	2.06E-05	2.29E-02	202936_s_at	NM_000346	SOX9
	
Male subjects					Male subjects				
	
1.21E-09	2.69E-05	206001_at	NM_000905	NPY	2.42E-10	5.40E-06	Drug use		
1.18E-08	1.31E-04	205984_at	NM_001882	CRHBP	1.21E-09	1.33E-05	206001_at	NM_000905	NPY
4.06E-08	3.01E-04	213921_at	NM_001048	SST	1.79E-09	1.33E-05	Alcohol use		
1.47E-07	8.22E-04	204326_x_at	NM_002450	MT1X	1.18E-08	6.56E-05	205984_at	NM_001882	CRHBP
1.98E-07	8.81E-04	221011_s_at	NM_030915	LBH	2.39E-08	1.06E-04	Brain pH		
3.41E-07	1.26E-03	208581_x_at	NM_005952	MT1X	4.06E-08	1.51E-04	213921_at	NM_001048	SST
8.38E-07	2.67E-03	217911_s_at	NM_004281	BAG3	1.47E-07	4.70E-04	204326_x_at	NM_002450	MT1X
1.11E-06	3.10E-03	202688_at	NM_003810	TNFSF10	1.98E-07	5.51E-04	221011_s_at	NM_030915	LBH
3.01E-06	7.27E-03	205336_at	NM_002854	PVALB	3.41E-07	8.44E-04	208581_x_at	NM_005952	MT1X
3.26E-06	7.27E-03	212859_x_at	BF217861	MT1E	8.38E-07	1.87E-03	217911_s_at	NM_004281	BAG3
3.72E-06	7.54E-03	209735_at	AF098951	ABCG2	1.11E-06	2.26E-03	202688_at	NM_003810	TNFSF10
4.18E-06	7.76E-03	220045_at	NM_022728	NEUROD6	3.01E-06	5.59E-03	205336_at	NM_002854	PVALB
5.00E-06	8.00E-03	221950_at	AI478455	EMX2	3.26E-06	5.59E-03	212859_x_at	BF217861	MT1E
5.03E-06	8.00E-03	202936_s_at	NM_000346	SOX9	3.72E-06	5.93E-03	209735_at	AF098951	ABCG2
5.41E-06	8.04E-03	211725_s_at	BC005884	BID	4.18E-06	6.21E-03	220045_at	NM_022728	NEUROD6
6.56E-06	8.98E-03	202071_at	NM_002999	SDC4	5.00E-06	6.59E-03	221950_at	AI478455	EMX2
6.85E-06	8.98E-03	212185_x_at	NM_005953	MT2A	5.03E-06	6.59E-03	202936_s_at	NM_000346	SOX9
8.18E-06	1.01E-02	202917_s_at	NM_002964	S100A8	5.41E-06	6.70E-03	211725_s_at	BC005884	BID
1.04E-05	1.22E-02	206461_x_at	NM_005951	MT1H/P2	6.56E-06	7.63E-03	202071_at	NM_002999	SDC4
1.27E-05	1.34E-02	206670_s_at	NM_013445	GAD1	6.85E-06	7.63E-03	212185_x_at	NM_005953	MT2A
	
Female subjects					Female subjects				
	
4.72E-04	1.00E+00	201041_s_at	NM_004417	DUSP1	4.72E-04	1.00E+00	201041_s_at	NM_004417	DUSP1
8.02E-04	1.00E+00	208078_s_at	NM_030751	SNF1LK	5.93E-04	1.00E+00	Age		
1.04E-03	1.00E+00	221841_s_at	BF514079	KLF4	8.02E-04	1.00E+00	208078_s_at	NM_030751	SNF1LK
1.35E-03	1.00E+00	201865_x_at	AI432196	NR3C1	1.04E-03	1.00E+00	221841_s_at	BF514079	KLF4
1.85E-03	1.00E+00	219044_at	NM_018271	THNSL2	1.35E-03	1.00E+00	201865_x_at	AI432196	NR3C1
2.53E-03	1.00E+00	202393_s_at	NM_005655	KLF10	1.85E-03	1.00E+00	219044_at	NM_018271	THNSL2
3.16E-03	1.00E+00	209189_at	BC004490	FOS	2.53E-03	1.00E+00	202393_s_at	NM_005655	KLF10
4.09E-03	1.00E+00	201417_at	AL136179	SOX4	2.81E-03	1.00E+00	Smoking at TOD		
6.61E-03	1.00E+00	211671_s_at	U01351	NR3C1	3.16E-03	1.00E+00	209189_at	BC004490	FOS
7.38E-03	1.00E+00	209457_at	U16996	DUSP5	4.09E-03	1.00E+00	201417_at	AL136179	SOX4
9.32E-03	1.00E+00	205856_at	NM_015865	SLC14A1	6.61E-03	1.00E+00	211671_s_at	U01351	NR3C1
9.73E-03	1.00E+00	213164_at	AI867198	SLC5A3	7.38E-03	1.00E+00	209457_at	U16996	DUSP5
1.24E-02	1.00E+00	214686_at	AA868898	ZNF266	9.32E-03	1.00E+00	205856_at	NM_015865	SLC14A1
1.26E-02	1.00E+00	201464_x_at	BG491844	JUN	9.73E-03	1.00E+00	213164_at	AI867198	SLC5A3
1.26E-02	1.00E+00	209900_s_at	AL162079	SLC16A1	1.24E-02	1.00E+00	214686_at	AA868898	ZNF266
1.28E-02	1.00E+00	205249_at	NM_000399	EGR2	1.26E-02	1.00E+00	201464_x_at	BG491844	JUN
1.39E-02	1.00E+00	200664_s_at	BG537255	DNAJB1	1.26E-02	1.00E+00	209900_s_at	AL162079	SLC16A1
1.62E-02	1.00E+00	202234_s_at	BF511091	SLC16A1	1.28E-02	1.00E+00	205249_at	NM_000399	EGR2
1.65E-02	1.00E+00	207547_s_at	NM_007177	FAM107A	1.39E-02	1.00E+00	200664_s_at	BG537255	DNAJB1
1.71E-02	1.00E+00	208691_at	BC001188	TFRC	1.62E-02	1.00E+00	202234_s_at	BF511091	SLC16A1

**Table 3:** Genes sorted by *p*-value, bipolar versus control

Expression data only					Demographic, clinical, and expression data				
	
All subjects					All subjects				
	
*p*-value	*q*-value	ID	GenBank	Symbol	*p*-value	*q*-value	ID	GenBank	Symbol
	
1.37E-09	2.19E-05	213921_at	NM_001048	SST	2.92E-18	4.66E-14	Drug use		
1.13E-06	5.75E-03	202688_at	NM_003810	TNFSF10	7.09E-13	5.66E-09	Alcohol use		
1.39E-06	5.75E-03	204185_x_at	NM_005038	PPID	1.37E-09	7.31E-06	213921_at	NM_001048	SST
1.44E-06	5.75E-03	208290_s_at	NM_001969	EIF5	1.13E-06	3.83E-03	202688_at	NM_003810	TNFSF10
2.48E-06	7.93E-03	210285_x_at	BC000383	WTAP	1.39E-06	3.83E-03	204185_x_at	NM_005038	PPID
3.16E-06	8.42E-03	220045_at	NM_022728	NEUROD6	1.44E-06	3.83E-03	208290_s_at	NM_001969	EIF5
3.88E-06	8.68E-03	211725_s_at	BC005884	BID	2.48E-06	5.67E-03	210285_x_at	BC000383	WTAP
4.35E-06	8.68E-03	208687_x_at	AF352832	HSPA8	3.16E-06	6.32E-03	220045_at	NM_022728	NEUROD6
5.27E-06	9.04E-03	212724_at	BG054844	RND3	3.88E-06	6.88E-03	211725_s_at	BC005884	BID
5.94E-06	9.04E-03	200881_s_at	NM_001539	DNAJA1	4.35E-06	6.94E-03	208687_x_at	AF352832	HSPA8
6.72E-06	9.04E-03	221011_s_at	NM_030915	LBH	5.27E-06	7.66E-03	212724_at	BG054844	RND3
6.89E-06	9.04E-03	203087_s_at	NM_004520	KIF2A	5.94E-06	7.84E-03	200881_s_at	NM_001539	DNAJA1
7.36E-06	9.04E-03	208708_x_at	AL080102	EIF5	6.72E-06	7.84E-03	221011_s_at	NM_030915	LBH
8.63E-06	9.85E-03	209619_at	K01144	CD74	6.89E-06	7.84E-03	203087_s_at	NM_004520	KIF2A
9.92E-06	1.01E-02	217932_at	NM_015971	MRPS7	7.36E-06	7.84E-03	208708_x_at	AL080102	EIF5
1.01E-05	1.01E-02	206001_at	NM_000905	NPY	8.63E-06	8.62E-03	209619_at	K01144	CD74
1.09E-05	1.01E-02	213038_at	AL031602	RNF19B	9.92E-06	9.00E-03	217932_at	NM_015971	MRPS7
1.15E-05	1.01E-02	204122_at	NM_003332	TYROBP	1.01E-05	9.00E-03	206001_at	NM_000905	NPY
1.20E-05	1.01E-02	212861_at	BF690150	MFSD5	1.09E-05	9.11E-03	213038_at	AL031602	RNF19B
1.47E-05	1.17E-02	211990_at	M27487	HLA-DPA1	1.15E-05	9.11E-03	204122_at	NM_003332	TYROBP
	
Male subjects					Male subjects				
	
5.38E-07	7.90E-03	202688_at	NM_003810	TNFSF10	1.68E-12	2.47E-08	Drug use		
1.14E-05	8.36E-02	210982_s_at	M60333	HLA-DRA	4.55E-10	3.34E-06	Alcohol use		
2.96E-05	1.08E-01	219525_at	NM_018242	SLC47A1	5.38E-07	2.63E-03	202688_at	NM_003810	TNFSF10
3.21E-05	1.08E-01	205859_at	NM_004271	LY86	1.14E-05	4.18E-02	210982_s_at	M60333	HLA-DRA
3.66E-05	1.08E-01	208894_at	M60334	HLA-DRA	2.96E-05	7.68E-02	219525_at	NM_018242	SLC47A1
6.47E-05	1.34E-01	204239_s_at	NM_005386	NNAT	3.21E-05	7.68E-02	205859_at	NM_004271	LY86
7.61E-05	1.34E-01	220045_at	NM_022728	NEUROD6	3.66E-05	7.68E-02	208894_at	M60334	HLA-DRA
8.23E-05	1.34E-01	213921_at	NM_001048	SST	6.47E-05	1.10E-01	204239_s_at	NM_005386	NNAT
8.23E-05	1.34E-01	201720_s_at	AI589086	LAPTM5	7.61E-05	1.10E-01	220045_at	NM_022728	NEUROD6
1.12E-04	1.65E-01	205984_at	NM_001882	CRHBP	8.23E-05	1.10E-01	213921_at	NM_001048	SST
1.37E-04	1.82E-01	204174_at	NM_001629	ALOX5AP	8.23E-05	1.10E-01	201720_s_at	AI589086	LAPTM5
1.57E-04	1.82E-01	209619_at	K01144	CD74	1.12E-04	1.38E-01	205984_at	NM_001882	CRHBP
1.61E-04	1.82E-01	204981_at	NM_002555	SLC22A18	1.37E-04	1.55E-01	204174_at	NM_001629	ALOX5AP
1.83E-04	1.92E-01	205404_at	NM_005525	HSD11B1	1.57E-04	1.58E-01	209619_at	K01144	CD74
1.99E-04	1.95E-01	204122_at	NM_003332	TYROBP	1.61E-04	1.58E-01	204981_at	NM_002555	SLC22A18
2.84E-04	2.61E-01	211991_s_at	M27487	HLA-DPA1	1.83E-04	1.68E-01	205404_at	NM_005525	HSD11B1
3.06E-04	2.65E-01	204670_x_at	NM_002125	HLA-DRB1	1.99E-04	1.68E-01	204122_at	NM_003332	TYROBP
4.02E-04	3.23E-01	220052_s_at	NM_012461	TINF2	2.06E-04	1.68E-01	Brain pH		
4.18E-04	3.23E-01	206001_at	NM_000905	NPY	2.84E-04	2.20E-01	211991_s_at	M27487	HLA-DPA1
4.56E-04	3.26E-01	207238_s_at	NM_002838	PTPRC	3.06E-04	2.25E-01	204670_x_at	NM_002125	HLA-DRB1
	
Female subjects					Female subjects				
	
4.27E-05	3.21E-01	221911_at	BE881590	ETV1	3.19E-08	3.84E-04	Drug use		
1.55E-04	3.21E-01	217828_at	NM_024755	SLTM	6.64E-06	4.00E-02	Age		
1.55E-04	3.21E-01	200881_s_at	NM_001539	DNAJA1	4.27E-05	1.71E-01	221911_at	BE881590	ETV1
1.72E-04	3.21E-01	201170_s_at	NM_003670	BHLHB2	1.55E-04	3.00E-01	217828_at	NM_024755	SLTM
1.74E-04	3.21E-01	217741_s_at	AW471220	ZFAND5	1.55E-04	3.00E-01	200881_s_at	NM_001539	DNAJA1
2.52E-04	3.21E-01	212724_at	BG054844	RND3	1.72E-04	3.00E-01	201170_s_at	NM_003670	BHLHB2
2.54E-04	3.21E-01	212514_x_at	R60068	DDX3X	1.74E-04	3.00E-01	217741_s_at	AW471220	ZFAND5
3.26E-04	3.21E-01	206302_s_at	NM_019094	NUDT4(P1)	2.52E-04	3.21E-01	212724_at	BG054844	RND3
3.34E-04	3.21E-01	208891_at	BC003143	DUSP6	2.54E-04	3.21E-01	212514_x_at	R60068	DDX3X
4.26E-04	3.21E-01	208687_x_at	AF352832	HSPA8	3.09E-04	3.21E-01	Left brain		
4.28E-04	3.21E-01	203087_s_at	NM_004520	KIF2A	3.26E-04	3.21E-01	206302_s_at	NM_019094	NUDT4(P1)
5.96E-04	3.21E-01	208893_s_at	BC005047	DUSP6	3.34E-04	3.21E-01	208891_at	BC003143	DUSP6
6.16E-04	3.21E-01	210285_x_at	BC000383	WTAP	4.26E-04	3.21E-01	208687_x_at	AF352832	HSPA8
6.22E-04	3.21E-01	208852_s_at	AI761759	CANX	4.28E-04	3.21E-01	203087_s_at	NM_004520	KIF2A
6.78E-04	3.21E-01	208892_s_at	BC003143	DUSP6	5.96E-04	3.21E-01	208893_s_at	BC005047	DUSP6
6.82E-04	3.21E-01	205251_at	NM_022817	PER2	6.16E-04	3.21E-01	210285_x_at	BC000383	WTAP
7.09E-04	3.21E-01	200033_at	NM_004396	DDX5	6.22E-04	3.21E-01	208852_s_at	AI761759	CANX
7.33E-04	3.21E-01	201604_s_at	NM_002480	PPP1R12A	6.78E-04	3.21E-01	208892_s_at	BC003143	DUSP6
7.45E-04	3.21E-01	204185_x_at	NM_005038	PPID	6.82E-04	3.21E-01	205251_at	NM_022817	PER2
7.52E-04	3.21E-01	204547_at	NM_006822	RAB40B	7.09E-04	3.21E-01	200033_at	NM_004396	DDX5

Comparing (a) the rankings for expression data only (the left panels of the tables) to (b) the rankings for expression and demographic data (the right panels) shows that similar features appear in (a) and (b). For example, all probe sets that appear in (b) also appear in (a) for Table 2, all subjects. In addition, (b) also includes a number of highly ranked demographic and clinical features. Table 2 shows, for example, that drug use and alcohol use are ranked high for the all and male subjects cases. This indicates that some of the demographic and clinical features are important to the classification tasks. Note that we also observed this while comparing classification models: the models with demographic and clinical features are more accurate.

When comparing the features that appear in the different tables, we observe that for the schizophrenia versus control task (Table 2, expression data), the rankings for all subjects and male subjects have 14 features in common: LBH [GenBank:NM_030915], MT1X [GenBank:NM_002450], MT1X [GenBank:NM_005952], TNFSF10 [GenBank:NM_003810], ABCG2 [GenBank:AF098951], MT1E [GenBank:BF217861], SST [GenBank:NM_001048], CRHBP [GenBank:NM_001882], EMX2 [GenBank:AI478455], NPY [GenBank:NM_000905], MT2A [GenBank:NM_005953], MT1H/P2 [GenBank:NM_005951], SOX9 [GenBank:NM_000346], S100A8 [GenBank:NM_002964].

On the other hand, the rankings for all subjects and female subjects have only one feature (FAM107A [GenBank:NM_007177]) in common. For the bipolar versus control task (Table 3, expression data) all subjects and male subjects share 6 features (SST [GenBank:NM_001048], TNFSF10 [GenBank:NM_003810], NEUROD6 [GenBank:NM_022728], CD74 [GenBank:K01144], NPY [GenBank:NM_000905], TYROBP [GenBank:NM_003332]), and all subjects and female subjects also share 6 features (PPID [GenBank:NM_005038], WTAP [GenBank:BC000383], RND3 [GenBank:BG054844], DNAJA1 [GenBank:NM_001539], KIF2A [GenBank:NM_004520]). For both diseases, there is no overlap between the ranking for the female subjects and that for the male subjects. Possibly of higher interest are the features relevant to both the schizophrenia versus control and bipolar versus control tasks. Comparing the rankings (the top left rankings of Table 2 and Table 3) shows that there are 4 common features: LBH [GenBank:NM_030915], TNFSF10 [GenBank:NM_003810], SST [GenBank:NM_001048], and NPY [GenBank:NM_000905]. These are relevant to both diseases.

Table 4 and Table 5 show rankings based on SVM weights. Also here the relevant features observed for expression and demographic data are similar to those found for expression data only. There is also overlap between the rankings for the different subject subsets (all, male only, and female only). Note, however, that the features identified with the SVM weights are different from those identified with the *p*-value method. Consider the expression data only, all subjects rankings. For schizophrenia versus control, there are no common features in the rankings produced by the *p*-value method (Table 2) and the SVM method (Table 4). For bipolar versus control (Tables 3 and 5), there are two shared features: SST [GenBank:NM_001048] and LBH [GenBank:NM_030915]. This difference in rankings arises because the methods essentially have a different goal: the *p*-value method looks for individual features that distinguish the two classes while the SVM method yields a set of features that together distinguish the classes.

**Table 4:** Genes sorted by SVM weight, schizophrenia versus control

Expression data only						Demographic, clinical, and expression data					
	
All subjects						All subjects					
	
SVM-weight	*p*-value	*q*-value	ID	GenBank	Symbol	SVM-weight	*p*-value	*q*-value	ID	GenBank	Symbol
	
-6.70E-02	1.13E-03	2.46E-01	201137_s_at	NM_002121	HLA-DPB1	1.06E-01	1.39E-10	3.11E-06	Drug use		
5.99E-02	4.71E-02	1.00E+00	214877_at	BE794663	CDKAL1	6.74E-02	2.88E-09	3.21E-05	Alcohol use		
-5.62E-02	1.19E-01	1.00E+00	218948_at	AL136679	QRSL1	5.16E-02	4.71E-02	1.00E+00	214877_at	BE794663	CDKAL1
-5.50E-02	5.31E-05	4.79E-02	203851_at	NM_002178	IGFBP6	-5.07E-02	5.31E-05	4.30E-02	203851_at	NM_002178	IGFBP6
-5.29E-02	6.22E-03	5.62E-01	204545_at	NM_000287	PEX6	-5.05E-02	6.22E-03	5.51E-01	204545_at	NM_000287	PEX6
5.21E-02	1.17E-01	1.00E+00	202944_at	NM_000262	NAGA	-4.84E-02	1.19E-01	1.00E+00	218948_at	AL136679	QRSL1
-4.83E-02	7.19E-02	1.00E+00	201123_s_at	NM_001970	EIF5A	4.60E-02	1.17E-01	1.00E+00	202944_at	NM_000262	NAGA
4.79E-02	7.06E-02	1.00E+00	210075_at	AF151074	2-Mar	4.57E-02	9.84E-02	1.00E+00	206785_s_at	NM_002260	KLRC2/1
4.76E-02	7.70E-02	1.00E+00	204418_x_at	NM_000848	GSTM2	-4.56E-02	1.13E-03	2.35E-01	201137_s_at	NM_002121	HLA-DPB1
4.76E-02	6.82E-02	1.00E+00	204550_x_at	NM_000561	GSTM1	-4.55E-02	5.69E-02	1.00E+00	218055_s_at	NM_018268	WDR41
-4.64E-02	9.14E-02	1.00E+00	218051_s_at	NM_022908	NT5DC2	4.51E-02	7.06E-02	1.00E+00	210075_at	AF151074	2-Mar
-4.56E-02	6.35E-02	1.00E+00	218002_s_at	NM_004887	CXCL14	-4.38E-02	5.28E-02	1.00E+00	219592_at	NM_024596	MCPH1
4.52E-02	9.84E-02	1.00E+00	206785_s_at	NM_002260	KLRC2/1	-4.31E-02	4.12E-02	1.00E+00	205145_s_at	NM_002477	MYL5
-4.48E-02	9.45E-02	1.00E+00	204295_at	NM_003172	SURF1	-4.23E-02	2.78E-02	1.00E+00	206108_s_at	NM_006275	SFRS6
-4.47E-02	2.78E-02	1.00E+00	206108_s_at	NM_006275	SFRS6	4.15E-02	6.82E-02	1.00E+00	204550_x_at	NM_000561	GSTM1
4.45E-02	3.91E-03	4.65E-01	212854_x_at	AB051480	NBPF10	4.14E-02	3.91E-03	4.54E-01	212854_x_at	AB051480	NBPF10
-4.37E-02	5.28E-02	1.00E+00	219592_at	NM_024596	MCPH1	-4.04E-02	6.35E-02	1.00E+00	218002_s_at	NM_004887	CXCL14
4.34E-02	1.10E-01	1.00E+00	201141_at	NM_002510	GPNMB	-3.98E-02	7.19E-02	1.00E+00	201123_s_at	NM_001970	EIF5A
4.32E-02	2.48E-02	1.00E+00	215823_x_at	U64661	LOC341315	3.96E-02	7.62E-06	1.13E-02	Smoking at TOD		
4.28E-02	4.33E-02	1.00E+00	221752_at	AL041728	SSH1	3.93E-02	2.48E-02	9.94E-01	215823_x_at	U64661	LOC341315
	
Male subjects						Male subjects					
	
-5.90E-02	1.59E-04	4.87E-02	201137_s_at	NM_002121	HLA-DPB1	7.25E-02	2.42E-10	5.40E-06	Drug use		
-5.81E-02	8.14E-02	9.87E-01	218948_at	AL136679	QRSL1	6.57E-02	1.79E-09	1.33E-05	Alcohol use		
-5.36E-02	2.46E-02	6.09E-01	218055_s_at	NM_018268	WDR41	-6.16E-02	3.87E-04	8.24E-02	HSV 1 OD Z-score		
4.73E-02	7.55E-03	3.68E-01	215009_s_at	U92014	SEC31A	-5.44E-02	2.46E-02	6.04E-01	218055_s_at	NM_018268	WDR41
3.97E-02	7.06E-02	9.39E-01	209823_x_at	M17955	HLA-DQB1	-4.98E-02	8.14E-02	9.84E-01	218948_at	AL136679	QRSL1
3.97E-02	3.96E-03	2.69E-01	220313_at	NM_022049	GPR88	4.71E-02	7.55E-03	3.64E-01	215009_s_at	U92014	SEC31A
3.90E-02	2.96E-02	6.65E-01	204550_x_at	NM_000561	GSTM1	-4.11E-02	1.59E-04	4.68E-02	201137_s_at	NM_002121	HLA-DPB1
3.64E-02	6.72E-02	9.24E-01	214877_at	BE794663	CDKAL1	4.03E-02	4.45E-04	8.62E-02	Smoking at TOD		
-3.63E-02	9.60E-05	3.77E-02	203851_at	NM_002178	IGFBP6	3.97E-02	3.96E-03	2.64E-01	220313_at	NM_022049	GPR88
-3.59E-02	8.41E-02	9.94E-01	221875_x_at	AW514210	HLA-F	3.67E-02	5.43E-02	8.51E-01	203554_x_at	NM_004219	PTTG1
3.58E-02	2.52E-02	6.15E-01	201141_at	NM_002510	GPNMB	-3.43E-02	8.71E-03	3.84E-01	204670_x_at	NM_002125	HLA-DRB1
-3.58E-02	1.57E-02	4.94E-01	203031_s_at	NM_000375	UROS	-3.35E-02	8.41E-02	9.90E-01	221875_x_at	AW514210	HLA-F
-3.56E-02	1.26E-02	4.60E-01	204295_at	NM_003172	SURF1	-3.32E-02	1.57E-02	4.90E-01	203031_s_at	NM_000375	UROS
3.56E-02	5.43E-02	8.55E-01	203554_x_at	NM_004219	PTTG1	-3.31E-02	9.60E-05	3.58E-02	203851_at	NM_002178	IGFBP6
3.55E-02	4.40E-02	7.84E-01	206339_at	NM_004291	CARTPT	3.26E-02	4.40E-02	7.80E-01	206339_at	NM_004291	CARTPT
-3.52E-02	9.35E-02	1.00E+00	208729_x_at	D83043	HLA-B	3.25E-02	7.06E-02	9.36E-01	209823_x_at	M17955	HLA-DQB1
-3.50E-02	8.71E-03	3.88E-01	204670_x_at	NM_002125	HLA-DRB1	-3.24E-02	9.35E-02	1.00E+00	208729_x_at	D83043	HLA-B
3.48E-02	6.76E-02	9.25E-01	221752_at	AL041728	SSH1	3.21E-02	6.76E-02	9.21E-01	221752_at	AL041728	SSH1
-3.46E-02	7.98E-02	9.82E-01	203374_s_at	AW612376	TPP2	3.16E-02	6.18E-02	8.93E-01	204570_at	NM_001864	COX7A1
3.38E-02	6.18E-02	8.97E-01	204570_at	NM_001864	COX7A1	3.09E-02	2.96E-02	6.61E-01	204550_x_at	NM_000561	GSTM1
	
Female subjects						Female subjects					
	
2.54E-02	1.85E-03	1.00E+00	219044_at	NM_018271	THNSL2	3.49E-02	2.07E-02	1.00E+00	Left brain		
-2.18E-02	1.71E-02	1.00E+00	208691_at	BC001188	TFRC	2.44E-02	1.85E-03	1.00E+00	219044_at	NM_018271	THNSL2
-2.12E-02	2.36E-01	1.00E+00	202747_s_at	NM_004867	ITM2A	-2.08E-02	1.71E-02	1.00E+00	208691_at	BC001188	TFRC
-2.04E-02	1.75E-01	1.00E+00	200606_at	NM_004415	DSP	2.07E-02	5.93E-04	1.00E+00	Age		
-2.03E-02	2.05E-01	1.00E+00	204305_at	NM_005932	MIPEP	-2.03E-02	2.36E-01	1.00E+00	202747_s_at	NM_004867	ITM2A
-2.01E-02	6.29E-02	1.00E+00	207332_s_at	NM_003234	TFRC	-1.99E-02	1.75E-01	1.00E+00	200606_at	NM_004415	DSP
-1.92E-02	5.61E-02	1.00E+00	202746_at	AL021786	ITM2A	-1.96E-02	2.05E-01	1.00E+00	204305_at	NM_005932	MIPEP
-1.86E-02	2.60E-02	1.00E+00	220576_at	NM_024989	PGAP1	-1.91E-02	6.29E-02	1.00E+00	207332_s_at	NM_003234	TFRC
1.79E-02	1.69E-01	1.00E+00	209619_at	K01144	CD74	-1.82E-02	5.61E-02	1.00E+00	202746_at	AL021786	ITM2A
1.77E-02	1.23E-01	1.00E+00	220954_s_at	NM_013440	PILRB	-1.78E-02	2.60E-02	1.00E+00	220576_at	NM_024989	PGAP1
-1.75E-02	1.35E-03	1.00E+00	201865_x_at	AI432196	NR3C1	1.76E-02	1.23E-01	1.00E+00	220954_s_at	NM_013440	PILRB
-1.73E-02	9.17E-02	1.00E+00	209735_at	AF098951	ABCG2	1.69E-02	1.69E-01	1.00E+00	209619_at	K01144	CD74
-1.71E-02	2.97E-01	1.00E+00	209314_s_at	AK024258	HBS1L	1.68E-02	8.54E-02	1.00E+00	Drug use		
-1.71E-02	5.99E-02	1.00E+00	209267_s_at	AB040120	SLC39A8	-1.66E-02	1.35E-03	1.00E+00	201865_x_at	AI432196	NR3C1
-1.70E-02	2.88E-01	1.00E+00	218051_s_at	NM_022908	NT5DC2	-1.65E-02	9.17E-02	1.00E+00	209735_at	AF098951	ABCG2
-1.66E-02	1.83E-01	1.00E+00	213791_at	NM_006211	PENK	-1.62E-02	5.99E-02	1.00E+00	209267_s_at	AB040120	SLC39A8
-1.66E-02	1.75E-01	1.00E+00	203697_at	U91903	FRZB	-1.61E-02	1.75E-01	1.00E+00	203697_at	U91903	FRZB
-1.63E-02	9.02E-02	1.00E+00	202688_at	NM_003810	TNFSF10	-1.61E-02	2.88E-01	1.00E+00	218051_s_at	NM_022908	NT5DC2
-1.62E-02	2.61E-01	1.00E+00	217757_at	NM_000014	A2M	-1.55E-02	1.83E-01	1.00E+00	213791_at	NM_006211	PENK
-1.60E-02	6.61E-03	1.00E+00	211671_s_at	U01351	NR3C1	-1.55E-02	3.39E-01	1.00E+00	222274_at	AW975050	FLJ31568

**Table 5:** Genes sorted by SVM weight, bipolar versus control

Expression data only						Demographic, clinical, and expression data					
	
All subjects						All subjects					
	
SVM-weight	*p*-value	*q*-value	ID	GenBank	Symbol	SVM-weight	*p*-value	*q*-value	ID	GenBank	Symbol
	
-1.04E-01	4.47E-03	9.53E-02	205033_s_at	NM_004084	DEFA1/3	2.00E-01	2.92E-18	4.66E-14	Drug use		
-1.03E-01	1.78E-03	6.98E-02	218055_s_at	NM_018268	WDR41	1.68E-01	7.09E-13	5.66E-09	Alcohol use		
-9.48E-02	1.34E-02	1.46E-01	203231_s_at	AW235612	ATXN1	-9.09E-02	3.22E-04	3.62E-02	202203_s_at	NM_001144	AMFR
-9.06E-02	1.70E-02	1.63E-01	219525_at	NM_018242	SLC47A1	-8.38E-02	4.47E-03	9.46E-02	205033_s_at	NM_004084	DEFA1/3
8.92E-02	9.79E-03	1.27E-01	215147_at	AF007147	CUGBP2	-7.98E-02	1.34E-02	1.45E-01	203231_s_at	AW235612	ATXN1
-8.86E-02	3.22E-04	3.72E-02	202203_s_at	NM_001144	AMFR	7.67E-02	6.28E-05	1.96E-02	PMI		
7.65E-02	2.36E-02	1.90E-01	204285_s_at	AI857639	PMAIP1	7.63E-02	9.79E-03	1.27E-01	215147_at	AF007147	CUGBP2
-7.61E-02	1.44E-03	6.36E-02	203031_s_at	NM_000375	UROS	6.58E-02	2.36E-02	1.89E-01	204285_s_at	AI857639	PMAIP1
-7.44E-02	6.61E-03	1.09E-01	218951_s_at	NM_018390	PLCXD1	-6.40E-02	1.78E-03	6.88E-02	218055_s_at	NM_018268	WDR41
7.17E-02	6.47E-03	1.09E-01	215528_at	AL049390	MGAT5	-6.23E-02	1.70E-02	1.62E-01	219525_at	NM_018242	SLC47A1
-6.98E-02	1.54E-03	6.46E-02	221579_s_at	AF062530	NUDT3	-5.94E-02	1.05E-02	1.32E-01	209189_at	BC004490	FOS
-6.67E-02	1.37E-09	2.19E-05	213921_at	NM_001048	SST	5.60E-02	6.47E-03	1.08E-01	215528_at	AL049390	MGAT5
-6.62E-02	1.05E-02	1.33E-01	209189_at	BC004490	FOS	-5.49E-02	6.61E-03	1.08E-01	218951_s_at	NM_018390	PLCXD1
-6.48E-02	6.72E-06	9.04E-03	221011_s_at	NM_030915	LBH	-5.46E-02	1.37E-09	7.31E-06	213921_at	NM_001048	SST
6.46E-02	4.79E-03	9.73E-02	217617_at	AW451711	PBX1	-5.44E-02	2.53E-03	7.69E-02	203528_at	NM_006378	SEMA4D
-5.98E-02	4.63E-03	9.59E-02	204507_s_at	NM_000945	PPP3R1	-5.40E-02	1.54E-03	6.37E-02	221579_s_at	AF062530	NUDT3
-5.87E-02	1.26E-03	6.14E-02	204545_at	NM_000287	PEX6	-5.33E-02	2.49E-03	7.69E-02	200976_s_at	NM_006024	TAX1BP1
5.80E-02	2.72E-02	2.02E-01	217482_at	AK021987	HEMBB1000354	5.33E-02	4.79E-03	9.66E-02	217617_at	AW451711	PBX1
-5.67E-02	2.53E-03	7.78E-02	203528_at	NM_006378	SEMA4D	-5.23E-02	1.26E-03	6.05E-02	204545_at	NM_000287	PEX6
5.45E-02	1.45E-02	1.52E-01	217055_x_at	S83374	SLC1A2	5.17E-02	1.45E-02	1.52E-01	217055_x_at	S83374	SLC1A2
	
Male subjects						Male subjects					
	
-8.15E-02	2.96E-05	1.08E-01	219525_at	NM_018242	SLC47A1	1.61E-01	4.55E-10	3.34E-06	Alcohol use		
-6.62E-02	2.50E-03	3.49E-01	208151_x_at	NM_030881	DDX17	1.27E-01	1.68E-12	2.47E-08	Drug use		
-6.36E-02	1.02E-02	3.49E-01	218055_s_at	NM_018268	WDR41	7.30E-02	3.77E-03	3.48E-01	PMI		
-6.35E-02	2.91E-03	3.49E-01	205048_s_at	NM_003832	PSPH	-6.30E-02	2.96E-05	7.68E-02	219525_at	NM_018242	SLC47A1
-6.12E-02	2.15E-03	3.49E-01	218948_at	AL136679	QRSL1	-5.73E-02	2.91E-03	3.48E-01	205048_s_at	NM_003832	PSPH
-5.44E-02	2.09E-03	3.49E-01	202203_s_at	NM_001144	AMFR	-5.47E-02	3.85E-03	3.48E-01	205924_at	BC005035	RAB3B
5.33E-02	4.53E-03	3.49E-01	216006_at	AF070620	WIPF2	-5.16E-02	2.50E-03	3.48E-01	208151_x_at	NM_030881	DDX17
-5.09E-02	3.85E-03	3.49E-01	205924_at	BC005035	RAB3B	-5.14E-02	2.09E-03	3.48E-01	202203_s_at	NM_001144	AMFR
-4.90E-02	1.17E-02	3.49E-01	208719_s_at	U59321	DDX17	-5.12E-02	2.15E-03	3.48E-01	218948_at	AL136679	QRSL1
4.79E-02	1.23E-02	3.49E-01	210738_s_at	AF011390	SLC4A4	-4.36E-02	1.02E-02	3.48E-01	218055_s_at	NM_018268	WDR41
4.79E-02	4.39E-02	3.49E-01	202853_s_at	NM_002958	RYK	4.20E-02	4.53E-03	3.48E-01	216006_at	AF070620	WIPF2
4.76E-02	2.39E-02	3.49E-01	207181_s_at	NM_001227	CASP7	-3.96E-02	2.06E-04	1.68E-01	Brain pH		
-4.74E-02	2.04E-02	3.49E-01	204416_x_at	NM_001645	APOC1	-3.84E-02	1.17E-02	3.48E-01	208719_s_at	U59321	DDX17
4.53E-02	1.52E-03	3.49E-01	204712_at	NM_007191	WIF1	3.76E-02	2.06E-02	3.48E-01	214722_at	AW516297	NOTCH2NL
-4.51E-02	3.53E-03	3.49E-01	204545_at	NM_000287	PEX6	3.75E-02	4.39E-02	3.48E-01	202853_s_at	NM_002958	RYK
-4.50E-02	6.16E-03	3.49E-01	205033_s_at	NM_004084	DEFA1/3	3.74E-02	9.42E-03	3.48E-01	209291_at	AW157094	ID4
4.31E-02	2.46E-02	3.49E-01	219255_x_at	NM_018725	IL17RB	-3.67E-02	2.04E-02	3.48E-01	204416_x_at	NM_001645	APOC1
-4.29E-02	8.23E-05	1.34E-01	213921_at	NM_001048	SST	-3.60E-02	3.53E-03	3.48E-01	204545_at	NM_000287	PEX6
4.26E-02	3.48E-02	3.49E-01	218500_at	NM_016647	C8orf55	3.56E-02	1.23E-02	3.48E-01	210738_s_at	AF011390	SLC4A4
-4.25E-02	5.38E-07	7.90E-03	202688_at	NM_003810	TNFSF10	-3.55E-02	6.16E-03	3.48E-01	205033_s_at	NM_004084	DEFA1/3
	
Female subjects						Female subjects					
	
6.17E-02	3.51E-02	3.21E-01	211751_at	BC005949	PDE4DIP	9.50E-02	3.19E-08	3.84E-04	Drug use		
-5.56E-02	3.95E-02	3.21E-01	202688_at	NM_003810	TNFSF10	8.85E-02	3.09E-04	3.21E-01	Left brain		
-4.20E-02	1.30E-02	3.21E-01	201222_s_at	AL527365	RAD23B	5.17E-02	3.51E-02	3.21E-01	211751_at	BC005949	PDE4DIP
-4.19E-02	1.89E-02	3.21E-01	211990_at	M27487	HLA-DPA1	5.01E-02	6.64E-06	4.00E-02	Age		
-4.04E-02	1.00E-02	3.21E-01	202581_at	NM_005346	HSPA1B	-4.93E-02	3.95E-02	3.21E-01	202688_at	NM_003810	TNFSF10
-3.77E-02	5.25E-02	3.21E-01	202203_s_at	NM_001144	AMFR	4.09E-02	2.66E-03	3.21E-01	Alcohol use		
-3.74E-02	4.26E-02	3.21E-01	217757_at	NM_000014	A2M	-3.88E-02	1.30E-02	3.21E-01	201222_s_at	AL527365	RAD23B
3.72E-02	5.74E-02	3.21E-01	213757_at	AA393940	EIF5A	-3.71E-02	1.00E-02	3.21E-01	202581_at	NM_005346	HSPA1B
-3.70E-02	2.24E-03	3.21E-01	221579_s_at	AF062530	NUDT3	-3.50E-02	5.25E-02	3.21E-01	202203_s_at	NM_001144	AMFR
-3.62E-02	1.15E-03	3.21E-01	203416_at	NM_000560	CD53	3.46E-02	5.74E-02	3.21E-01	213757_at	AA393940	EIF5A
-3.49E-02	2.55E-03	3.21E-01	208691_at	BC001188	TFRC	-3.29E-02	4.26E-02	3.21E-01	217757_at	NM_000014	A2M
3.36E-02	2.92E-03	3.21E-01	205990_s_at	NM_003392	WNT5A	-3.26E-02	1.15E-03	3.21E-01	203416_at	NM_000560	CD53
-3.33E-02	2.68E-02	3.21E-01	202291_s_at	NM_000900	MGP	-3.25E-02	1.89E-02	3.21E-01	211990_at	M27487	HLA-DPA1
-3.32E-02	2.63E-03	3.21E-01	200800_s_at	NM_005345	HSPA1A/B	-2.99E-02	2.68E-02	3.21E-01	202291_s_at	NM_000900	MGP
-3.30E-02	2.60E-02	3.21E-01	211038_s_at	BC006312	CROCCL1	-2.95E-02	2.24E-03	3.21E-01	221579_s_at	AF062530	NUDT3
-3.27E-02	4.16E-02	3.21E-01	218589_at	NM_005767	P2RY5	-2.91E-02	2.63E-03	3.21E-01	200800_s_at	NM_005345	HSPA1A/B
-3.23E-02	6.22E-02	3.21E-01	203231_s_at	AW235612	ATXN1	2.89E-02	2.92E-03	3.21E-01	205990_s_at	NM_003392	WNT5A
-3.21E-02	5.41E-02	3.21E-01	218055_s_at	NM_018268	WDR41	-2.89E-02	1.45E-02	3.21E-01	202746_at	AL021786	ITM2A
-3.17E-02	3.12E-02	3.21E-01	210004_at	AF035776	OLR1	-2.79E-02	5.14E-02	3.21E-01	209458_x_at	AF105974	HBA1/2
-3.11E-02	1.70E-02	3.21E-01	206577_at	NM_003381	VIP	-2.75E-02	6.56E-02	3.21E-01	201718_s_at	BF511685	EPB41L2

### Biological relevance

Of the top 20 genes identified using the *p*-value ranking, 11 have been previously implicated in schizophrenia in at least one study. These genes include: NR4A3 [GenBank:U12767] [16-19], SST [GenBank:NM_001048] [20], NPY [GenBank:NM_000905] [21,22], S100A8 [GenBank:NM_002964] [23], CRH [GenBank:NM_000756] [24,25], GAD1 [GenBank:NM_013445] [26,27], FOS [GenBank:BC004490] [28,29], JUN [GenBank:BG491844] [28,29], DNAJB1 [GenBank:BG537255] [30], SLC16A1 [GenBank:AL162079, GenBank:BF511091] [31], and EGR2 [GenBank:NM_000399] [32,17].

Overlap with the current literature occurs for bipolar disorder as well, although overlap is not as large primarily because of the relative immaturity of the field and concomitant smaller number of literature results. Of the top 20 genes identified using SVM weight or *p*-value, 7 genes have been implicated previously in bipolar disorder. Interestingly, multiple probes for the same gene are in the top 20 for DUSP6 [GenBank:BC003143, GenBank:BC005047] [33,34] and HLA-DRA [GenBank:M60333, GenBank:M60334] [18]. Single probes previously implicated in bipolar disorder include: SST [GenBank:NM_001048] [20], HLA-A [GenBank:AA573862] [35], NPY [GenBank:NM_000905] [36], HLA-DRB3 [Genbank:NM_002125] [37], and DNAJB1 [GenBank:BG537255] [30].

**Figure 15:**
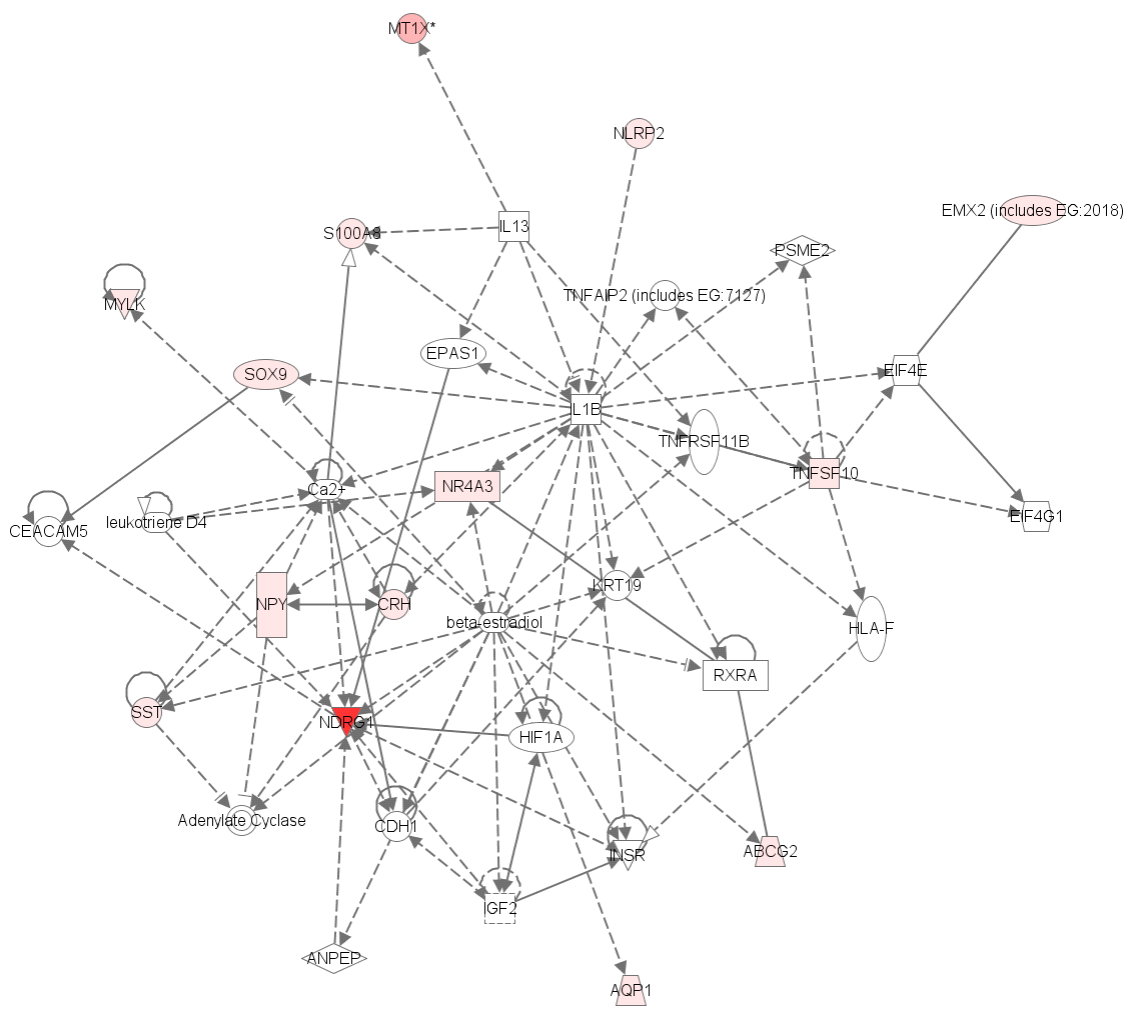
**Biological network representing the schizophrenia *p*-value ranking**. The network was generated using Ingenuity Systems Pathway analysis. The darker the red the more significant the correlation with the disease.

**Figure 16:**
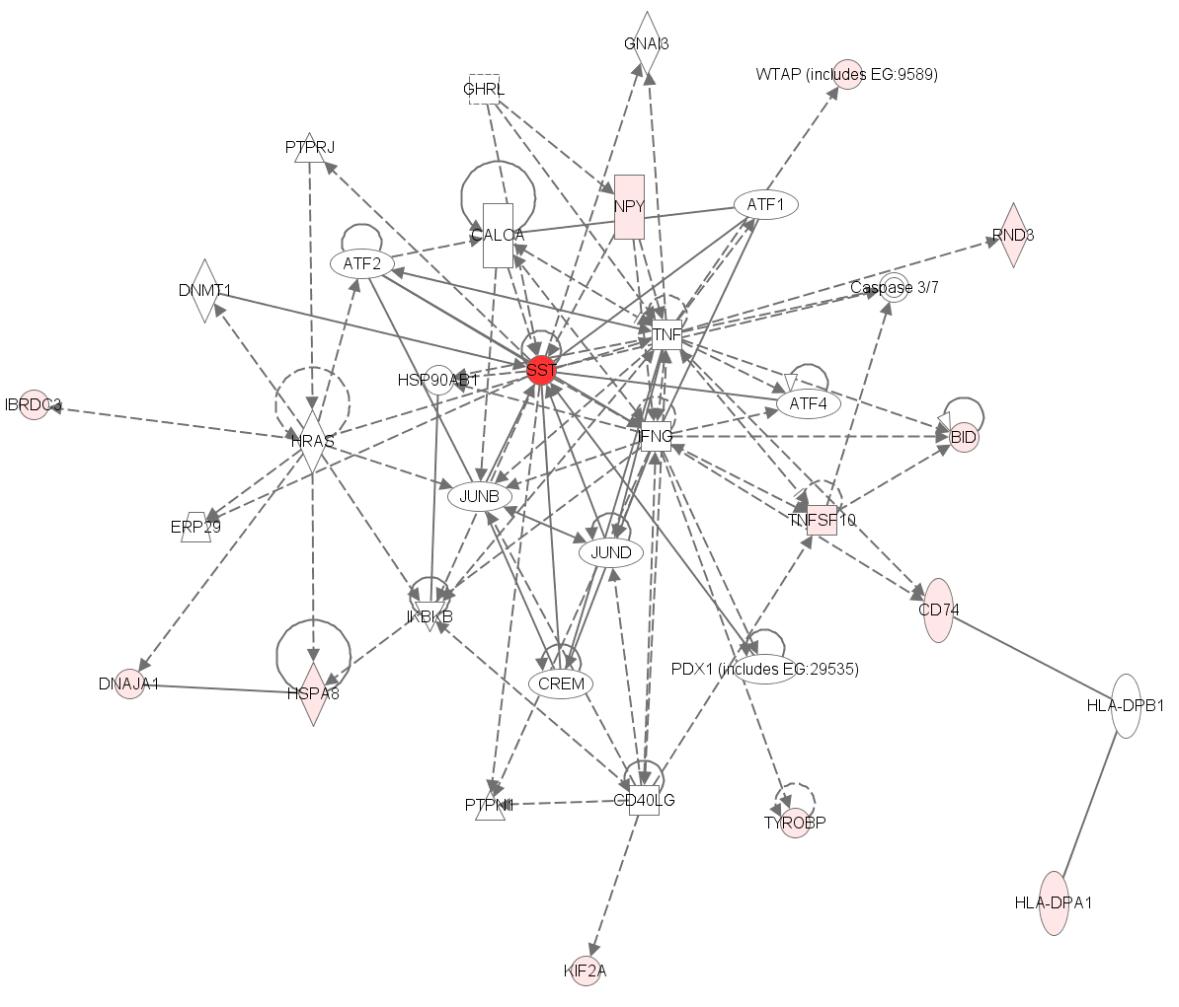
**Biological network representing the bipolar disorder *p*-value ranking**. The network was generated using Ingenuity Systems Pathway analysis. The darker the red the more significant the correlation with the disease.

Interestingly, most of the remaining genes in the list are known to interact with the genes that have a documented association with either bipolar disorder or schizophrenia. These interactions were determined using Ingenuity Systems software. 14 of the 20 genes in the schizophrenia sample are involved in the same biological pathway (Figure 15). By combining the two networks generated by the software package via 3 overlapping genes, 19 of the 20 genes are in a single biological network. Similarly, 13 of the 20 genes are in a single pathway for bipolar disorder (Figure 16). By combining two of the 3 generated pathways through 3 overlapping genes, this biological network represents 16 of the 20 genes on the list.

One of the more remarkable features of this analysis is the difference in gene expression patterns between males and females. Much speculation has surrounded the role of gender in psychiatric disorders based on morphological and clinical comparisons between affected males and affected females. This analysis may provide further evidence to support and broaden this hypothesis. The most prevalent gender-based differences associated with mental disorders are in the structural abnormalities that have long been known in schizophrenia [38]. These have been validated using CT and MRI scans, demonstrating differences in ventricle size in males and females with schizophrenia; specifically the left ventricles of males are known to be enlarged relative to both their healthy counterparts and affected females [39-45]. Another structure showing a difference in affected males and females is the corpus calosum [46-48]. The temporal lobe appears smaller in affected men than women [49]. Specifically, the superior temporal gyri [50], the posterior superior temporal gyrus [51], and Herschel's gyri [51] have all been shown in one or more studies to be reduced in affected males when compared to their unaffected male counterparts or affected females. Volume reductions have also been observed in the amygdale-hipocampal complex [52]. Reduced asymmetry of the planum temporale has been observed in females in both MRI and post mortem studies [53-56]. In this study we provide additional evidence to further bolster the claim of gender differences, but this new evidence is in the form of molecular differences between affected males and females in both schizophrenia and bipolar disorder. This may all provide evidence that gender may have confounded the results of past molecular analyses into these disorders.

The ranking based on the SVM weights does not produce a significant number of genes previously implicated in schizophrenia or bipolar disorder. This does not necessarily mean this measure does not provide as much biological insight as the ranking based on *p*-value. The smaller overlap may instead be because the SVM-based method is more different from previous studies than is the method based on *p*-values. Whereas previous studies sought individual genes, much as the ranking by *p*-value does, the ranking by SVM weight seeks genes that are predictive in the context of other genes. Therefore it seems likely that this more global look at bipolar disorder and schizophrenia is producing genes missed in previous analyses of microarray data on brain tissue.

### Impact of alcohol and drug use

Consider the schizophrenia versus control classification task. Feature rankings by *p*-value, such as Table 2, may include alcohol use (AU) and drug use (DU) associated genes, and some of these may not be associated to schizophrenia. AU and DU are known to alter gene expression, that is, there are genes that are differently expressed in heavy AU/DU subjects and in low AU/DU subjects. Such AU/DU associated genes will also be differently expressed in the schizophrenia and control classes, simply because there are more high AU/DU subjects in the schizophrenia class, and more low AU/DU subjects in the control class (Table 1). Therefore, AU/DU associated genes may appear in the feature rankings (Table 2).

Identical reasoning applies to the bipolar disorder versus control task, which exhibits a similar difference in AU/DU distribution between the two classes. This should be kept in mind when analyzing the rankings. Note that these differences in distribution are already present in the population and therefore difficult to avoid in the samples.

Post-stratification can potentially be used to remove the confounding effects of variables such as AU and DU. Essentially, post-stratification computes a subset of the data such that the subset's AU/DU distribution is identical in each class. We have applied post-stratification. A detailed description of the method that we used together with its results is available in additional files 1 and 2. In these results however, post-stratification proved ineffective because it significantly reduced the amount of data, and therefore also the power of the statistical methods, resulting in unacceptably high false discovery rates. We briefly quantify this in the following paragraph.

Consider the *p*-value ranking for the schizophrenia versus control task, expression data only, all subjects (Table 2). Reporting all top 20 features as being significant results in a false discovery rate of 3.8%, that is, fewer than one feature is a false discovery. However, if we compute a similar ranking on the stratified data, which contains only 121 of the 332 samples in the original data , then reporting only the four top-ranked features as significant already yields a false discovery rate of 62.8%, that is, more than two of these four may be false discoveries. Because of this high false discovery rate, we decided not to use stratification in the paper and to accept the possibility of AU/DU associated genes in the rankings. Further analysis, possibly using more data, is required to identify such genes.

Note that this problem is partially mitigated by use of the SVM-based ranking instead, when using demographic features in addition to gene expression. If a gene's correlation with disease is only because of its correlations with AU/DU, then the SVM will prefer to place most/all of the weight on AU and DU rather than on this gene. The gene will receive high weight only if it provides additional predictive ability for disease beyond its association with AU/DU. As a result, the ranking will mostly include genes that are truly associated to the disease, which may explain the difference between the SVM and *p*-value rankings. The extent of this mitigation requires further study to quantify, which is difficult because we do not have a ground truth to compare to, that is, it would require that we know which genes are directly associated with AU/DU and are not associated with the diseases.

## Conclusion

This paper demonstrates that both bipolar disorder and schizophrenia induce substantial changes in gene expression within the brain – substantial enough that each can be distinguished from normal control with an area under the ROC curve of over 0.9. The paper also demonstrates the utility of combining gene expression and clinical data. To our knowledge, this is the first time such a combination has been employed on this scale. Finally, the paper demonstrates the significant advantage of support vector machines for this task over other widely used algorithms from statistical classification and machine learning.

Using these classification schemes we have shown an overlap with the current literature when ranking the genes according to *p*-value. In fact, nearly the entire schizophrenia and the entire bipolar disorder list are either indicated in the literature or are involved in a biological network with a previously implicated gene. However, when ranking the genes based on SVM weight only 1 or 2 genes out of 20 on each list overlapped with the current literature. This does not necessarily imply that these methods are not viable but rather that these may be previously unidentified candidates that have risen to the top due to the large sample size of this analysis and the application of alternative classification algorithms for microarray data analysis.

This paper also discussed the possible impact of variables such as alcohol and drug use on the presented gene rankings. Post-stratification can correct for such variables, but it significantly reduces the power of the statistical methods. Therefore, data for more controls with high values for these variables is required so that the AU/DU distributions in the different classes become more similar. It should also be kept in mind that this is a retrospective study on a given data set and that all results will require further clinical validation. Samples in the collection were matched during the collection process for as many parameters as possible. While there may be some confounding effects from pre-mortem consumption of alcohol and other non-prescription medication, this analysis is our best attempt to account for differences in the factors through analytical means (Torrey, Webster, et al 2000). Since, these samples are not derived from controlled animals models we will have to rely on these analytical means to aid our efforts in dissecting the root causes of complex diseases.

## Methods

### Encoding of nominal features

Because most classification techniques that we consider are restricted to numerical features only, we re-encode each nominal feature as a numeric feature. Table 1 indicates the encoding after each feature value. For the binary features "Sex", "Left brain", "Brain region", and "Smoking at time of death", we use an encoding with three values: 1, 0, and -1, with 0 indicating "unknown". The features that have essentially ordered values, such as "Alcohol use", "Drug use" and "Rate of death", we encode with a simple integer encoding.

### Software packages and versions

We choose the SVM-light software version 6.01 [57] for constructing linear soft margin SVMs. We use the NSC software PAM version 1.28, which is available at: . We use the reimplementation of C4.5 that is available in the Weka data mining tool version 3.4.4 [58]. We also use Weka 3.4.4's implementations of NB and *k*NN. We use the EOV software version 1.0 by Hardin et al. [9]. To compute the *q*-values, we use the software QVALUE version 1.1 developed by Storey [15]. All are freely available.

### ROC curves and AUC

Most classifiers provide confidence scores for their predictions. The classification behavior of such classifiers can be modified by applying a threshold to this score: only predict positive if the confidence is above the given threshold. By varying the threshold, we obtain different ROC points, which can be connected into a curve. (The curve can then be used, for example, to select an appropriate threshold.) We present such a curve for each classifier and report the corresponding AUC.

To obtain the ROC curves, we use 10-fold cross-validation (CV). 10-fold CV is often used to evaluate the predictive performance of classifiers if the number of instances is small. In this situation, 10-fold CV results in a lower-variance estimate of error than does the use of a single held-aside test set. 10-fold CV consists of three steps: (a) partition the data set *D *into 10 subsets *T*_*i*_; (b) train 10 classifiers on the training sets *D-T*_*i*_; (c) test classifier *i *on test set *T*_*i*_. We pool the predicted confidence values of the classifiers over the 10 test sets to construct the ROC curve. The CV algorithm that we employ is stratified, which means that it ensures that the *T*_*i *_have identical class distributions, or as nearly identical as possible.

To assess statistical significance when comparing two classification techniques by AUC value, we use a two-sided paired *t*-test. The paired sample values used in the test are the AUC values computed for the two techniques on the 10 CV test sets.

### Algorithm parameters

Most classification techniques come with a number of parameters. We set all parameters to their default values, except for the following. The parameter *C *of SVM-light, which controls the contribution of the misclassified examples, is set to 1.0 (SVM-light is not particularly sensitive to *C*'s value). We enable Laplace smoothing of DT's confidence values. Following Hardin et al. [9], we set EOV's number of decision stumps *N *to 20. We enable Weka's discretization feature for NB. We run *k*NN with *k *= 3 neighbors.

We tune NSC's Δ parameter, which controls the amount of shrinkage, by means of 10-fold CV, as suggested by Tibshirani et al. [7]. Recall that we also create ROC curves by means of CV. To avoid overfitting the test set, we repeat the parameter tuning each time we run NSC, that is, for each ROC CV fold. The tuning selects the value of Δ that maximizes TP + TN, with TP the true positive rate and TN the proportion of negative examples that are correctly classified.

The performance of some classification techniques can be improved by running a feature selection method prior to constructing the classifier. We implement feature selection for SVM, NB, and 3NN. The feature selection works as follows. We perform a two-sided paired *t*-test for each feature comparing its value in the two classes. Then we rank the features by their *t*-test's *p*-value and retain the 10% features that most significantly differ in the two classes (similar to the *p*-value ranking discussed before). We repeat the feature selection for each CV fold and perform the *t*-test on the corresponding training set.

To compute the feature ranking by SVM weight, we normalize each feature by subtracting its mean and dividing by its standard deviation (in the data set at hand), and run the SVM algorithm on the transformed data. The rationale behind this is to avoid favoring features with a small value range in the ranking. We also enable feature selection to construct the ranking by SVM weight as discussed before.

## Authors' contributions

JS performed the statistical analysis and drafted the manuscript. SD provided the data, provided critical input and drafted the biological relevance section of the manuscript. DP supervised the study and helped drafting the manuscript. All authors read and approved the final manuscript.

## Description of additional data files

• FeatureRankingsStratified.{doc, pdf} (Microsoft Word and PDF format): "Feature rankings on post-stratified data" in additional files 1 and 2.

Feature rankings similar to Tables 2, 3, 4, 5 computed for a post-stratified copy of the data set. See Section "Impact of alcohol and drug use" for more information.

• FeatureRankingsDetailed.xls (Microsoft Excel format): "Feature rankings with additional information" in additional file 3.

Feature rankings that contain the same information as Tables 2, 3, 4, 5, but also include additional information, such as gene title and chromosome location.

## Supplementary Material

Additional file 1 **Feature rankings on post-stratified data** Click here for file

Additional file 2 **Feature rankings on post-stratified data** Click here for file

Additional file 3 Feature rankings with additional information Click here for file
