# Supplementary material for: Combining gene expression, demographic and clinical data in modeling disease: a case study of bipolar disorder and schizophrenia
Source: BMC Genomics. 2008 Nov 7;9:531. doi: 10.1186/1471-2164-9-531 (PMC2628394; doi:10.1186/1471-2164-9-531)
Supplement: Additional file 1 — Feature rankings on post-stratified data [file 1471-2164-9-531-S1.doc]

## Feature rankings on post-stratified data

## Method

The goal of the stratification is to obtain an identical distribution of the features ‘alcohol use’ (AU) and ‘drug use’ (DU) in the six different data sets that we consider: male-control, male-schiz., male-bipolar, female-control, female-schiz., and female-bipolar. We take as target the marginal distributions of AU and DU in the set of all controls: *P_AU_(C)* and *P_DU_(C)*, with *C* the set of controls. Then we determine, for each of the six data sets in turn, the subset *S_i_* of samples of which the AU and DU marginals (*P_AU_(S_i_)* and *P_DU_(S_i_)*) best approximate the corresponding target marginals. To compare the marginal AU/DU distribution of a subset to the target marginal, we employ KL-divergence (*d_KL_*), a measure often used to compare probability distributions. In particular, the stratification procedure selects a subset *S_i_* that minimizes *d_KL_(P_AU_(C), P_AU_(S_i_ )) + d_KL_(P_DU_(C), P_DU_(S_i_))* and that contains at least 33% of the data set’s samples (with a minimum of 15 samples). We choose this lower bound because beyond this point SVM’s performance drops significantly and becomes close to random guessing. Using more samples, on the other hand, results in a much higher KL-divergence: the control AU distribution is particularly difficult to obtain in the male-bipolar subset, where alcohol usage is biased to higher values. With this trade-off between KL-divergence and number of samples, the resulting stratified data set $\bigcup_{i} S_{i}$contains 121 samples (the original data contains 332 samples). Figure 1 shows histograms of the AU/DU marginals in the different stratified data sets. The feature rankings by *p*-value and SVM weight for the stratified data can be found in the next few pages.

Figure 1: Result after stratification. Each of the six subsets: male-control, male-schiz., male-bipolar, female-control, female-schiz., and female-bipolar has been sub-sampled to mimic the distribution of alcohol and drug use in the overall control class (inidicated in red).

Genes sorted by *p*-value, schizophrenia versus control (stratified data)

Genes sorted by *p*-value, bipolar versus control (stratified data)

Genes sorted by SVM-weight, schizophrenia versus control (stratified data)

Genes sorted by SVM-weight, bipolar versus control (stratified data)
