# Supplementary material for: Combining gene expression, demographic and clinical data in modeling disease: a case study of bipolar disorder and schizophrenia
Source: BMC Genomics. 2008 Nov 7;9:531. doi: 10.1186/1471-2164-9-531 (PMC2628394; doi:10.1186/1471-2164-9-531)
Supplement: Additional file 2 — Feature rankings on post-stratified data [file 1471-2164-9-531-S2.pdf]

## Feature rankings on post-stratified data

### Method

The goal of the stratification is to obtain an identical distribution of the features 'alcohol use' (AU) and 'drug use' (DU) in the six different data sets that we consider: male-control, male-schiz., male-bipolar, female-control, female-schiz., and female-bipolar. We take as target the marginal distributions of AU and DU in the set of all controls: $P_{AU}(C)$ and $P_{DU}(C)$, with $C$ the set of controls. Then we determine, for each of the six data sets in turn, the subset $S_i$ of samples of which the AU and DU marginals ($P_{AU}(S_i)$ and $P_{DU}(S_i)$) best approximate the corresponding target marginals. To compare the marginal AU/DU distribution of a subset to the target marginal, we employ KL-divergence ($d_{KL}$), a measure often used to compare probability distributions. In particular, the stratification procedure selects a subset $S_i$ that minimizes $d_{KL}(P_{AU}(C), P_{AU}(S_i)) + d_{KL}(P_{DU}(C), P_{DU}(S_i))$ and that contains at least 33% of the data set's samples (with a minimum of 15 samples). We choose this lower bound because beyond this point SVM's performance drops significantly and becomes close to random guessing. Using more samples, on the other hand, results in a much higher KL-divergence: the control AU distribution is particularly difficult to obtain in the male-bipolar subset, where alcohol usage is biased to higher values. With this trade-off between KL-divergence and number of samples, the resulting stratified data set $\bigcup_i S_i$ contains 121 samples (the original data contains 332 samples). Figure 1 shows histograms of the AU/DU marginals in the different stratified data sets. The feature rankings by *p*-value and SVM weight for the stratified data can be found in the next few pages.

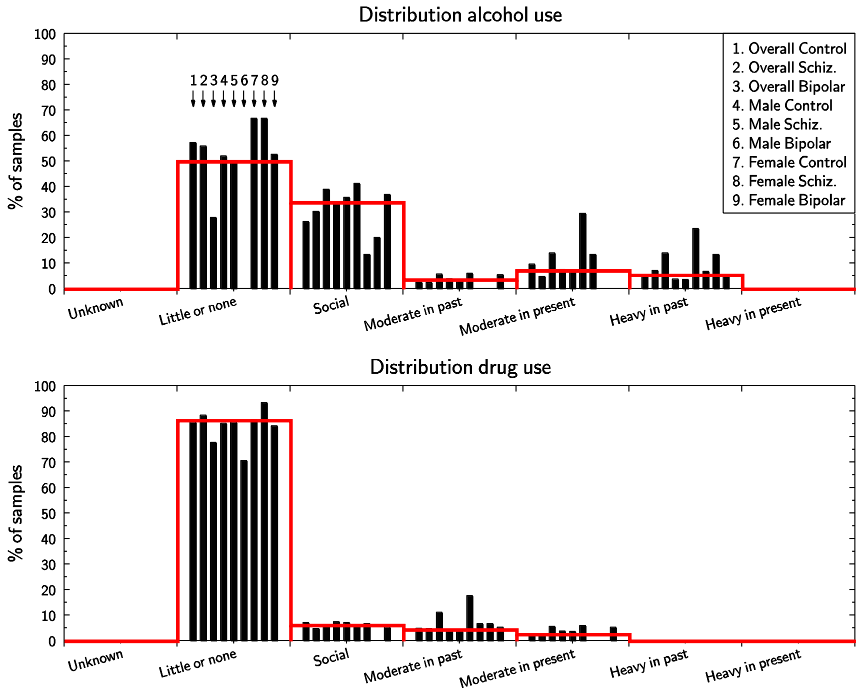


**Figure 1: Result after stratification. Each of the six subsets: male-control, male-schiz., male-bipolar, female-control, female-schiz., and female-bipolar has been sub-sampled to mimic the distribution of alcohol and drug use in the overall control class (inidicated in red).**

Genes sorted by *p*-value, schizophrenia versus control (stratified data)

| Expression data only | | | | | Demographic + expression data | | | | |
|---|---|---|---|---|---|---|---|---|---|
| **All subjects** | | | | | **All subjects** | | | | |
| *p-value* | *q-value* | ID | GenBank | Symbol | *p-value* | *q-value* | ID | GenBank | Symbol |
| 5.42E-06 | 8.85E-02 | 221011_s_at | NM_030915 | LBH | 5.42E-06 | 8.85E-02 | 221011_s_at | NM_030915 | LBH |
| 7.94E-06 | 8.85E-02 | 203349_s_at | NM_004454 | ETV5 | 7.94E-06 | 8.85E-02 | 203349_s_at | NM_004454 | ETV5 |
| 5.74E-05 | 4.27E-01 | 213921_at | NM_001048 | SST | 5.74E-05 | 4.27E-01 | 213921_at | NM_001048 | SST |
| 1.13E-04 | 6.28E-01 | 202393_s_at | NM_005655 | KLF10 | 1.13E-04 | 6.28E-01 | 202393_s_at | NM_005655 | KLF10 |
| 1.59E-04 | 7.11E-01 | 205630_at | NM_000756 | CRH | 1.59E-04 | 7.11E-01 | 205630_at | NM_000756 | CRH |
| 2.04E-04 | 7.57E-01 | 208892_s_at | BC003143 | DUSP6 | 2.04E-04 | 7.57E-01 | 208892_s_at | BC003143 | DUSP6 |
| 2.78E-04 | 8.11E-01 | 213793_s_at | BE550452 | HOMER1 | 2.78E-04 | 8.12E-01 | 213793_s_at | BE550452 | HOMER1 |
| 2.97E-04 | 8.11E-01 | 202478_at | NM_021643 | TRIB2 | 2.97E-04 | 8.12E-01 | 202478_at | NM_021643 | TRIB2 |
| 3.28E-04 | 8.11E-01 | 214655_at | U18549 | GPR6 | 3.28E-04 | 8.12E-01 | 214655_at | U18549 | GPR6 |
| 3.78E-04 | 8.42E-01 | 220313_at | NM_022049 | GPR88 | 3.78E-04 | 8.42E-01 | 220313_at | NM_022049 | GPR88 |
| 7.49E-04 | 1.00E+00 | 205856_at | NM_015865 | SLC14A1 | 7.49E-04 | 1.00E+00 | 205856_at | NM_015865 | SLC14A1 |
| 8.91E-04 | 1.00E+00 | 217667_at | AV761014 | LOC729799 | 8.42E-04 | 1.00E+00 | Rate of death | | |
| 9.21E-04 | 1.00E+00 | 208891_at | BC003143 | DUSP6 | 8.91E-04 | 1.00E+00 | 217667_at | AV761014 | LOC729799 |
| 1.34E-03 | 1.00E+00 | 207285_x_at | NM_001318 | CSHL1 | 9.21E-04 | 1.00E+00 | 208891_at | BC003143 | DUSP6 |
| 1.38E-03 | 1.00E+00 | 213900_at | AA524029 | C9orf61 | 1.34E-03 | 1.00E+00 | 207285_x_at | NM_001318 | CSHL1 |
| 1.58E-03 | 1.00E+00 | 220675_s_at | NM_025225 | PNPLA3 | 1.38E-03 | 1.00E+00 | 213900_at | AA524029 | C9orf61 |
| 1.61E-03 | 1.00E+00 | 208893_s_at | BC005047 | DUSP6 | 1.58E-03 | 1.00E+00 | 220675_s_at | NM_025225 | PNPLA3 |
| 1.82E-03 | 1.00E+00 | 206282_at | NM_002500 | NEUROD1 | 1.61E-03 | 1.00E+00 | 208893_s_at | BC005047 | DUSP6 |
| 2.05E-03 | 1.00E+00 | 219230_at | NM_018286 | TMEM100 | 1.82E-03 | 1.00E+00 | 206282_at | NM_002500 | NEUROD1 |
| 2.18E-03 | 1.00E+00 | 208687_x_at | AF352832 | HSPA8,… | 2.05E-03 | 1.00E+00 | 219230_at | NM_018286 | TMEM100 |
| **Male subjects** | | | | | **Male subjects** | | | | |
| 3.01E-05 | 6.60E-01 | 213921_at | NM_001048 | SST | 3.01E-05 | 6.61E-01 | 213921_at | NM_001048 | SST |
| 1.37E-04 | 9.83E-01 | 213793_s_at | BE550452 | HOMER1 | 1.37E-04 | 9.83E-01 | 213793_s_at | BE550452 | HOMER1 |
| 1.92E-04 | 9.83E-01 | 206001_at | NM_000905 | NPY | 1.92E-04 | 9.83E-01 | 206001_at | NM_000905 | NPY |
| 2.53E-04 | 9.83E-01 | 203349_s_at | NM_004454 | ETV5 | 2.53E-04 | 9.83E-01 | 203349_s_at | NM_004454 | ETV5 |
| 3.19E-04 | 9.83E-01 | 205630_at | NM_000756 | CRH | 3.19E-04 | 9.83E-01 | 205630_at | NM_000756 | CRH |
| 6.89E-04 | 9.83E-01 | 221011_s_at | NM_030915 | LBH | 6.89E-04 | 9.83E-01 | 221011_s_at | NM_030915 | LBH |
| 7.09E-04 | 9.83E-01 | 208892_s_at | BC003143 | DUSP6 | 7.09E-04 | 9.83E-01 | 208892_s_at | BC003143 | DUSP6 |
| 8.66E-04 | 9.83E-01 | 207768_at | NM_001965 | EGR4 | 8.66E-04 | 9.83E-01 | 207768_at | NM_001965 | EGR4 |
| 1.22E-03 | 9.83E-01 | 220313_at | NM_022049 | GPR88 | 1.22E-03 | 9.83E-01 | 220313_at | NM_022049 | GPR88 |
| 1.81E-03 | 9.83E-01 | 205547_s_at | NM_003186 | TAGLN | 1.81E-03 | 9.83E-01 | 205547_s_at | NM_003186 | TAGLN |
| 1.86E-03 | 9.83E-01 | 215009_s_at | U92014 | SEC31A | 1.86E-03 | 9.83E-01 | 215009_s_at | U92014 | SEC31A |
| 2.06E-03 | 9.83E-01 | 203233_at | NM_000418 | IL4R | 2.06E-03 | 9.83E-01 | 203233_at | NM_000418 | IL4R |
| 2.19E-03 | 9.83E-01 | 202917_s_at | NM_002964 | S100A8 | 2.19E-03 | 9.83E-01 | 202917_s_at | NM_002964 | S100A8 |
| 2.37E-03 | 9.83E-01 | 210381_s_at | BC000740 | CCKBR | 2.37E-03 | 9.83E-01 | 210381_s_at | BC000740 | CCKBR |
| 2.93E-03 | 9.83E-01 | 219230_at | NM_018286 | TMEM100 | 2.93E-03 | 9.83E-01 | 219230_at | NM_018286 | TMEM100 |
| 3.00E-03 | 9.83E-01 | 219313_at | NM_017577 | GRAMD1C | 3.00E-03 | 9.83E-01 | 219313_at | NM_017577 | GRAMD1C |
| 3.12E-03 | 9.83E-01 | 208891_at | BC003143 | DUSP6 | 3.12E-03 | 9.83E-01 | 208891_at | BC003143 | DUSP6 |
| 3.20E-03 | 9.83E-01 | 208159_x_at | NM_004399 | DDX11 | 3.20E-03 | 9.83E-01 | 208159_x_at | NM_004399 | DDX11 |
| 3.29E-03 | 9.83E-01 | 208451_s_at | NM_000592 | C4B/A | 3.29E-03 | 9.83E-01 | 208451_s_at | NM_000592 | C4B/A |
| 3.39E-03 | 9.83E-01 | 203336_s_at | AL548363 | ITGB1BP1 | 3.39E-03 | 9.83E-01 | 203336_s_at | AL548363 | ITGB1BP1 |
| **Female subjects** | | | | | **Female subjects** | | | | |
| 2.22E-03 | 1.00E+00 | 205726_at | NM_006729 | DIAPH2 | 2.22E-03 | 1.00E+00 | 205726_at | NM_006729 | DIAPH2 |
| 2.90E-03 | 1.00E+00 | 219036_at | NM_024491 | CEP70 | 2.90E-03 | 1.00E+00 | 219036_at | NM_024491 | CEP70 |
| 3.31E-03 | 1.00E+00 | 221011_s_at | NM_030915 | LBH | 3.31E-03 | 1.00E+00 | 221011_s_at | NM_030915 | LBH |
| 3.90E-03 | 1.00E+00 | 219044_at | NM_018271 | THNSL2 | 3.90E-03 | 1.00E+00 | 219044_at | NM_018271 | THNSL2 |
| 5.08E-03 | 1.00E+00 | 202393_s_at | NM_005655 | KLF10 | 5.08E-03 | 1.00E+00 | 202393_s_at | NM_005655 | KLF10 |
| 6.30E-03 | 1.00E+00 | 215866_at | AK024938 | CHCHD3 | 6.30E-03 | 1.00E+00 | 215866_at | AK024938 | CHCHD3 |
| 8.56E-03 | 1.00E+00 | 203551_s_at | NM_004375 | COX11 | 8.56E-03 | 1.00E+00 | 203551_s_at | NM_004375 | COX11 |
| 1.05E-02 | 1.00E+00 | 217667_at | AV761014 | LOC729799 | 1.05E-02 | 1.00E+00 | 217667_at | AV761014 | LOC729799 |
| 1.19E-02 | 1.00E+00 | 207285_x_at | NM_001318 | CSHL1 | 1.19E-02 | 1.00E+00 | 207285_x_at | NM_001318 | CSHL1 |
| 1.20E-02 | 1.00E+00 | 202437_s_at | NM_000104 | CYP1B1 | 1.20E-02 | 1.00E+00 | 202437_s_at | NM_000104 | CYP1B1 |
| 1.25E-02 | 1.00E+00 | 218945_at | NM_024109 | C16orf68 | 1.25E-02 | 1.00E+00 | 218945_at | NM_024109 | C16orf68 |
| 1.26E-02 | 1.00E+00 | 203349_s_at | NM_004454 | ETV5 | 1.26E-02 | 1.00E+00 | 203349_s_at | NM_004454 | ETV5 |
| 1.28E-02 | 1.00E+00 | 213675_at | W61005 | CBR01467 | 1.28E-02 | 1.00E+00 | 213675_at | W61005 | CBR01467 |
| 1.42E-02 | 1.00E+00 | 207381_at | NM_001139 | ALOX12B | 1.42E-02 | 1.00E+00 | 207381_at | NM_001139 | ALOX12B |
| 1.43E-02 | 1.00E+00 | 202478_at | NM_021643 | TRIB2 | 1.43E-02 | 1.00E+00 | 202478_at | NM_021643 | TRIB2 |
| 1.62E-02 | 1.00E+00 | 201865_x_at | AI432196 | NR3C1 | 1.62E-02 | 1.00E+00 | 201865_x_at | AI432196 | NR3C1 |
| 2.00E-02 | 1.00E+00 | 214087_s_at | BF593509 | MYBPC1 | 2.00E-02 | 1.00E+00 | 214087_s_at | BF593509 | MYBPC1 |
| 2.02E-02 | 1.00E+00 | 214968_at | AV694312 | DDX51 | 2.02E-02 | 1.00E+00 | 214968_at | AV694312 | DDX51 |
| 2.05E-02 | 1.00E+00 | 205769_at | NM_003645 | SLC27A2 | 2.05E-02 | 1.00E+00 | 205769_at | NM_003645 | SLC27A2 |
| 2.12E-02 | 1.00E+00 | 217996_at | AA576961 | PHLDA1 | 2.12E-02 | 1.00E+00 | 217996_at | AA576961 | PHLDA1 |

Genes sorted by *p*-value, bipolar versus control (stratified data)

| Expression data only | | | | | Demographic + expression data | | | | |
|---|---|---|---|---|---|---|---|---|---|
| All subjects | | | | | All subjects | | | | |
| *p-value* | *q-value* | ID | GenBank | Symbol | *p-value* | *q-value* | ID | GenBank | Symbol |
| 7.53E-07 | 8.06E-03 | 202203_s_at | NM_001144 | AMFR | 7.53E-07 | 8.07E-03 | 202203_s_at | NM_001144 | AMFR |
| 6.05E-06 | 2.93E-02 | 201170_s_at | NM_003670 | BHLHB2 | 6.05E-06 | 2.93E-02 | 201170_s_at | NM_003670 | BHLHB2 |
| 8.22E-06 | 2.93E-02 | 202478_at | NM_021643 | TRIB2 | 8.22E-06 | 2.93E-02 | 202478_at | NM_021643 | TRIB2 |
| 1.19E-05 | 3.14E-02 | 208938_at | BC004913 | PRCC | 1.19E-05 | 3.15E-02 | 208938_at | BC004913 | PRCC |
| 1.47E-05 | 3.14E-02 | 213921_at | NM_001048 | SST | 1.47E-05 | 3.15E-02 | 213921_at | NM_001048 | SST |
| 2.18E-05 | 3.89E-02 | 201865_x_at | AI432196 | NR3C1 | 2.18E-05 | 3.89E-02 | 201865_x_at | AI432196 | NR3C1 |
| 2.72E-05 | 4.16E-02 | 211671_s_at | U01351 | NR3C1 | 2.72E-05 | 4.16E-02 | 211671_s_at | U01351 | NR3C1 |
| 3.19E-05 | 4.27E-02 | 200709_at | NM_000801 | FKBP1A | 3.19E-05 | 4.28E-02 | 200709_at | NM_000801 | FKBP1A |
| 5.51E-05 | 6.55E-02 | 203946_s_at | U75667 | ARG2 | 5.51E-05 | 6.55E-02 | 203946_s_at | U75667 | ARG2 |
| 6.26E-05 | 6.70E-02 | 209357_at | AF109161 | CITED2 | 6.26E-05 | 6.70E-02 | 209357_at | AF109161 | CITED2 |
| 7.48E-05 | 7.27E-02 | 221428_s_at | NM_030921 | TBL1XR1 | 7.48E-05 | 7.28E-02 | 221428_s_at | NM_030921 | TBL1XR1 |
| 8.39E-05 | 7.27E-02 | 221760_at | BG287153 | MAN1A1 | 8.39E-05 | 7.28E-02 | 221760_at | BG287153 | MAN1A1 |
| 8.84E-05 | 7.27E-02 | 217767_at | NM_000064 | LOC653879 | 8.84E-05 | 7.28E-02 | 217767_at | NM_000064 | LOC653879 |
| 1.03E-04 | 7.50E-02 | 202479_s_at | BC002637 | TRIB2 | 1.03E-04 | 7.51E-02 | 202479_s_at | BC002637 | TRIB2 |
| 1.16E-04 | 7.50E-02 | 220333_at | NM_017705 | PAQR5 | 1.16E-04 | 7.51E-02 | 220333_at | NM_017705 | PAQR5 |
| 1.17E-04 | 7.50E-02 | 204185_x_at | NM_005038 | PPID | 1.17E-04 | 7.51E-02 | 204185_x_at | NM_005038 | PPID |
| 1.19E-04 | 7.50E-02 | 213338_at | BF062629 | TMEM158 | 1.19E-04 | 7.51E-02 | 213338_at | BF062629 | TMEM158 |
| 1.40E-04 | 8.02E-02 | 207980_s_at | NM_006079 | CITED2 | 1.40E-04 | 8.02E-02 | 207980_s_at | NM_006079 | CITED2 |
| 1.42E-04 | 8.02E-02 | 208893_s_at | BC005047 | DUSP6 | 1.42E-04 | 8.02E-02 | 208893_s_at | BC005047 | DUSP6 |
| 1.61E-04 | 8.12E-02 | 202675_at | NM_003000 | SDHB | 1.61E-04 | 8.12E-02 | 202675_at | NM_003000 | SDHB |
| Male subjects | | | | | Male subjects | | | | |
| 1.36E-05 | 2.73E-01 | 202203_s_at | NM_001144 | AMFR | 1.36E-05 | 2.73E-01 | 202203_s_at | NM_001144 | AMFR |
| 1.23E-03 | 8.72E-01 | 221489_s_at | W48843 | SPRY4 | 2.02E-04 | 8.72E-01 | Alcohol use | | |
| 1.74E-03 | 8.72E-01 | 213921_at | NM_001048 | SST | 1.23E-03 | 8.72E-01 | 221489_s_at | W48843 | SPRY4 |
| 1.80E-03 | 8.72E-01 | 208891_at | BC003143 | DUSP6 | 1.74E-03 | 8.72E-01 | 213921_at | NM_001048 | SST |
| 2.34E-03 | 8.72E-01 | 204122_at | NM_003332 | TYROBP | 1.80E-03 | 8.72E-01 | 208891_at | BC003143 | DUSP6 |
| 2.67E-03 | 8.72E-01 | 208893_s_at | BC005047 | DUSP6 | 2.34E-03 | 8.72E-01 | 204122_at | NM_003332 | TYROBP |
| 2.76E-03 | 8.72E-01 | 204239_s_at | NM_005386 | NNAT | 2.67E-03 | 8.72E-01 | 208893_s_at | BC005047 | DUSP6 |
| 2.90E-03 | 8.72E-01 | 217767_at | NM_000064 | LOC653879 | 2.76E-03 | 8.72E-01 | 204239_s_at | NM_005386 | NNAT |
| 3.49E-03 | 8.72E-01 | 203349_s_at | NM_004454 | ETV5 | 2.90E-03 | 8.72E-01 | 217767_at | NM_000064 | LOC653879 |
| 4.37E-03 | 8.72E-01 | 210090_at | AF193421 | ARC | 3.49E-03 | 8.72E-01 | 203349_s_at | NM_004454 | ETV5 |
| 4.41E-03 | 8.72E-01 | 201041_s_at | NM_004417 | DUSP1 | 4.37E-03 | 8.72E-01 | 210090_at | AF193421 | ARC |
| 4.70E-03 | 8.72E-01 | 205630_at | NM_000756 | CRH | 4.41E-03 | 8.72E-01 | 201041_s_at | NM_004417 | DUSP1 |
| 4.83E-03 | 8.72E-01 | 221011_s_at | NM_030915 | LBH | 4.70E-03 | 8.72E-01 | 205630_at | NM_000756 | CRH |
| 4.90E-03 | 8.72E-01 | 201170_s_at | NM_003670 | BHLHB2 | 4.83E-03 | 8.72E-01 | 221011_s_at | NM_030915 | LBH |
| 5.16E-03 | 8.72E-01 | 208892_s_at | BC003143 | DUSP6 | 4.90E-03 | 8.72E-01 | 201170_s_at | NM_003670 | BHLHB2 |
| 6.43E-03 | 8.72E-01 | 202478_at | NM_021643 | TRIB2 | 5.16E-03 | 8.72E-01 | 208892_s_at | BC003143 | DUSP6 |
| 7.88E-03 | 8.72E-01 | 204545_at | NM_000287 | PEX6 | 6.43E-03 | 8.72E-01 | 202478_at | NM_021643 | TRIB2 |
| 7.91E-03 | 8.72E-01 | 222117_s_at | AF131745 | ADCK2 | 7.88E-03 | 8.72E-01 | 204545_at | NM_000287 | PEX6 |
| 8.29E-03 | 8.72E-01 | 209738_x_at | M31125 | PSG6 | 7.91E-03 | 8.72E-01 | 222117_s_at | AF131745 | ADCK2 |
| 8.66E-03 | 8.72E-01 | 221178_at | NM_025045 | BAIAP2L2 | 8.29E-03 | 8.72E-01 | 209738_x_at | M31125 | PSG6 |
| Female subjects | | | | | Female subjects | | | | |
| 3.43E-05 | 3.18E-01 | 201865_x_at | AI432196 | NR3C1 | 1.72E-06 | 2.04E-02 | Age | | |
| 8.36E-05 | 3.18E-01 | 211671_s_at | U01351 | NR3C1 | 3.43E-05 | 2.04E-01 | 201865_x_at | AI432196 | NR3C1 |
| 1.46E-04 | 3.18E-01 | 221760_at | BG287153 | MAN1A1 | 8.36E-05 | 3.18E-01 | 211671_s_at | U01351 | NR3C1 |
| 1.97E-04 | 3.18E-01 | 213338_at | BF062629 | TMEM158 | 1.46E-04 | 3.18E-01 | 221760_at | BG287153 | MAN1A1 |
| 4.20E-04 | 3.18E-01 | 200881_s_at | NM_001539 | DNAJA1 | 1.97E-04 | 3.18E-01 | 213338_at | BF062629 | TMEM158 |
| 4.24E-04 | 3.18E-01 | 205037_at | NM_006860 | RABL4 | 4.20E-04 | 3.18E-01 | 200881_s_at | NM_001539 | DNAJA1 |
| 4.51E-04 | 3.18E-01 | 201170_s_at | NM_003670 | BHLHB2 | 4.24E-04 | 3.18E-01 | 205037_at | NM_006860 | RABL4 |
| 5.17E-04 | 3.18E-01 | 209538_at | U69645 | ZNF32 | 4.51E-04 | 3.18E-01 | 201170_s_at | NM_003670 | BHLHB2 |
| 5.23E-04 | 3.18E-01 | 207614_s_at | NM_003592 | CUL1 | 5.17E-04 | 3.18E-01 | 209538_at | U69645 | ZNF32 |
| 5.32E-04 | 3.18E-01 | 214359_s_at | AI218219 | HSP90AB1 | 5.23E-04 | 3.18E-01 | 207614_s_at | NM_003592 | CUL1 |
| 5.34E-04 | 3.18E-01 | 202478_at | NM_021643 | TRIB2 | 5.32E-04 | 3.18E-01 | 214359_s_at | AI218219 | HSP90AB1 |
| 5.43E-04 | 3.18E-01 | 221428_s_at | NM_030921 | TBL1XR1 | 5.34E-04 | 3.18E-01 | 202478_at | NM_021643 | TRIB2 |
| 5.79E-04 | 3.18E-01 | 212515_s_at | BG492602 | DDX3X | 5.43E-04 | 3.18E-01 | 221428_s_at | NM_030921 | TBL1XR1 |
| 5.84E-04 | 3.18E-01 | 200709_at | NM_000801 | FKBP1A | 5.79E-04 | 3.18E-01 | 212515_s_at | BG492602 | DDX3X |
| 6.43E-04 | 3.18E-01 | 208938_at | BC004913 | PRCC | 5.84E-04 | 3.18E-01 | 200709_at | NM_000801 | FKBP1A |
| 6.45E-04 | 3.18E-01 | 204185_x_at | NM_005038 | PPID | 6.43E-04 | 3.18E-01 | 208938_at | BC004913 | PRCC |
| 6.48E-04 | 3.18E-01 | 218559_s_at | NM_005461 | MAFB | 6.45E-04 | 3.18E-01 | 204185_x_at | NM_005038 | PPID |
| 6.89E-04 | 3.18E-01 | 206302_s_at | NM_019094 | NUDT4(P1) | 6.48E-04 | 3.18E-01 | 218559_s_at | NM_005461 | MAFB |
| 6.94E-04 | 3.18E-01 | 34031_i_at | U90269 | KRIT1 | 6.89E-04 | 3.18E-01 | 206302_s_at | NM_019094 | NUDT4(P1) |
| 7.05E-04 | 3.18E-01 | 208290_s_at | NM_001969 | EIF5 | 6.94E-04 | 3.18E-01 | 34031_i_at | U90269 | KRIT1 |

Genes sorted by SVM-weight, schizophrenia versus control (stratified data)

| Expression data only | | | | | | Demographic + expression data | | | | | |
|---|---|---|---|---|---|---|---|---|---|---|---|
| All subjects | | | | | | All subjects | | | | | |
| SVM-weight | *p* -value | *q* -value | ID | GenBank | Symbol | SVM-weight | *p* -value | *q* -value | ID | GenBank | Symbol |
| -4.47E-02 | 7.19E-03 | 1.00E+00 | 202688_at | NM_003810 | TNFSF10 | -4.45E-02 | 7.19E-03 | 1.00E+00 | 202688_at | NM_003810 | TNFSF10 |
| -3.64E-02 | 1.64E-01 | 1.00E+00 | 202295_s_at | NM_004390 | CTSH | -3.63E-02 | 1.64E-01 | 1.00E+00 | 202295_s_at | NM_004390 | CTSH |
| -3.40E-02 | 2.27E-02 | 1.00E+00 | 204416_x_at | NM_001645 | APOC1 | -3.38E-02 | 2.27E-02 | 1.00E+00 | 204416_x_at | NM_001645 | APOC1 |
| 3.34E-02 | 3.03E-02 | 1.00E+00 | 215009_s_at | U92014 | SEC31A | 3.34E-02 | 3.03E-02 | 1.00E+00 | 215009_s_at | U92014 | SEC31A |
| -3.27E-02 | 1.28E-01 | 1.00E+00 | 216967_at | AF279774 | GAP43 | -3.27E-02 | 1.28E-01 | 1.00E+00 | 216967_at | AF279774 | GAP43 |
| -3.08E-02 | 1.41E-01 | 1.00E+00 | 208396_s_at | NM_005019 | PDE1A | -3.06E-02 | 1.41E-01 | 1.00E+00 | 208396_s_at | NM_005019 | PDE1A |
| 3.02E-02 | 1.41E-02 | 1.00E+00 | 219044_at | NM_018271 | THNSL2 | 3.00E-02 | 1.41E-02 | 1.00E+00 | 219044_at | NM_018271 | THNSL2 |
| -2.95E-02 | 9.02E-02 | 1.00E+00 | 202687_s_at | U57059 | TNFSF10 | -2.94E-02 | 9.02E-02 | 1.00E+00 | 202687_s_at | U57059 | TNFSF10 |
| -2.93E-02 | 1.68E-01 | 1.00E+00 | 204304_s_at | NM_006017 | PROM1 | -2.94E-02 | 1.68E-01 | 1.00E+00 | 204304_s_at | NM_006017 | PROM1 |
| 2.90E-02 | 1.95E-01 | 1.00E+00 | 207227_x_at | NM_006605 | RFPL2/1/3 | 2.91E-02 | 1.95E-01 | 1.00E+00 | 207227_x_at | NM_006605 | RFPL2/1/3 |
| -2.73E-02 | 5.42E-06 | 8.85E-02 | 221011_s_at | NM_030915 | LBH | -2.70E-02 | 5.42E-06 | 8.85E-02 | 221011_s_at | NM_030915 | LBH |
| -2.66E-02 | 3.32E-02 | 1.00E+00 | 213791_at | NM_006211 | PENK | -2.66E-02 | 3.32E-02 | 1.00E+00 | 213791_at | NM_006211 | PENK |
| -2.55E-02 | 8.79E-02 | 1.00E+00 | 216231_s_at | AW188940 | B2M | 2.56E-02 | 1.23E-01 | 1.00E+00 | 205872_x_at | NM_022359 | PDE4DIP |
| -2.55E-02 | 1.24E-01 | 1.00E+00 | 208894_at | M60334 | HLA-DRA | -2.55E-02 | 8.79E-02 | 1.00E+00 | 216231_s_at | AW188940 | B2M |
| 2.55E-02 | 1.23E-01 | 1.00E+00 | 205872_x_at | NM_022359 | PDE4DIP | -2.54E-02 | 1.24E-01 | 1.00E+00 | 208894_at | M60334 | HLA-DRA |
| -2.50E-02 | 8.38E-02 | 1.00E+00 | 211528_x_at | M90685 | HLA-G | 2.50E-02 | 8.67E-02 | 1.00E+00 | 207693_at | NM_000726 | CACNB4 |
| 2.50E-02 | 8.67E-02 | 1.00E+00 | 207693_at | NM_000726 | CACNB4 | -2.50E-02 | 8.38E-02 | 1.00E+00 | 211528_x_at | M90685 | HLA-G |
| -2.46E-02 | 7.59E-02 | 1.00E+00 | 220276_at | NM_024730 | FLJ22655 | -2.46E-02 | 7.59E-02 | 1.00E+00 | 220276_at | NM_024730 | FLJ22655 |
| -2.46E-02 | 1.65E-02 | 1.00E+00 | 201865_x_at | AI432196 | NR3C1 | -2.45E-02 | 1.65E-02 | 1.00E+00 | 201865_x_at | AI432196 | NR3C1 |
| 2.43E-02 | 2.58E-03 | 1.00E+00 | 217336_at | AL118510 | LOC133569,… | 2.44E-02 | 2.58E-03 | 1.00E+00 | 217336_at | AL118510 | LOC133569,… |
| Male subjects | | | | | | Male subjects | | | | | |
| 3.50E-02 | 1.86E-03 | 9.83E-01 | 215009_s_at | U92014 | SEC31A | 3.48E-02 | 1.86E-03 | 9.83E-01 | 215009_s_at | U92014 | SEC31A |
| 3.12E-02 | 2.14E-02 | 9.83E-01 | 203337_x_at | NM_004763 | ITGB1BP1 | 3.06E-02 | 2.14E-02 | 9.83E-01 | 203337_x_at | NM_004763 | ITGB1BP1 |
| -2.59E-02 | 1.40E-02 | 9.83E-01 | 202688_at | NM_003810 | TNFSF10 | -2.59E-02 | 1.40E-02 | 9.83E-01 | 202688_at | NM_003810 | TNFSF10 |
| -2.59E-02 | 1.04E-01 | 9.83E-01 | 204670_x_at | NM_002125 | HLA-DRB1,… | -2.59E-02 | 1.04E-01 | 9.83E-01 | 204670_x_at | NM_002125 | HLA-DRB1,… |
| -2.49E-02 | 8.89E-03 | 9.83E-01 | 205586_x_at | NM_003378 | VGF | -2.49E-02 | 7.28E-02 | 9.83E-01 | 216967_at | AF279774 | GAP43 |
| -2.48E-02 | 7.28E-02 | 9.83E-01 | 216967_at | AF279774 | GAP43 | -2.46E-02 | 8.89E-03 | 9.83E-01 | 205586_x_at | NM_003378 | VGF |
| -2.39E-02 | 1.07E-01 | 9.83E-01 | 204416_x_at | NM_001645 | APOC1 | -2.38E-02 | 9.25E-02 | 9.83E-01 | 208894_at | M60334 | HLA-DRA |
| -2.39E-02 | 9.25E-02 | 9.83E-01 | 208894_at | M60334 | HLA-DRA | -2.37E-02 | 1.07E-01 | 9.83E-01 | 204416_x_at | NM_001645 | APOC1 |
| -2.27E-02 | 6.96E-03 | 9.83E-01 | 205984_at | NM_001882 | CRHBP | -2.27E-02 | 6.96E-03 | 9.83E-01 | 205984_at | NM_001882 | CRHBP |
| -2.25E-02 | 1.37E-04 | 9.83E-01 | 213793_s_at | BE550452 | HOMER1 | -2.24E-02 | 1.37E-04 | 9.83E-01 | 213793_s_at | BE550452 | HOMER1 |
| -2.19E-02 | 8.54E-02 | 9.83E-01 | 213979_s_at | BF984434 | CTBP1 | -2.20E-02 | 8.54E-02 | 9.83E-01 | 213979_s_at | BF984434 | CTBP1 |
| -2.18E-02 | 3.01E-05 | 6.60E-01 | 213921_at | NM_001048 | SST | -2.17E-02 | 3.01E-05 | 6.61E-01 | 213921_at | NM_001048 | SST |
| 2.13E-02 | 6.92E-02 | 9.83E-01 | 204805_s_at | NM_006026 | H1FX | 2.13E-02 | 6.92E-02 | 9.83E-01 | 204805_s_at | NM_006026 | H1FX |
| -2.06E-02 | 1.34E-01 | 9.83E-01 | 209619_at | K01144 | CD74 | -2.06E-02 | 4.33E-02 | 9.83E-01 | 209312_x_at | U65585 | HLA-DRB1,… |
| -2.06E-02 | 4.33E-02 | 9.83E-01 | 209312_x_at | U65585 | HLA-DRB1,… | -2.05E-02 | 1.34E-01 | 9.83E-01 | 209619_at | K01144 | CD74 |
| -2.04E-02 | 6.89E-04 | 9.83E-01 | 221011_s_at | NM_030915 | LBH | -2.03E-02 | 6.89E-04 | 9.83E-01 | 221011_s_at | NM_030915 | LBH |
| 2.01E-02 | 2.93E-03 | 9.83E-01 | 219230_at | NM_018286 | TMEM100 | 2.00E-02 | 2.93E-03 | 9.83E-01 | 219230_at | NM_018286 | TMEM100 |
| -2.00E-02 | 6.39E-03 | 9.83E-01 | 209735_at | AF098951 | ABCG2 | -2.00E-02 | 6.39E-03 | 9.83E-01 | 209735_at | AF098951 | ABCG2 |
| -1.97E-02 | 2.37E-02 | 9.83E-01 | 201694_s_at | NM_001964 | EGR1 | -1.97E-02 | 9.23E-03 | 9.83E-01 | 202393_s_at | NM_005655 | KLF10 |
| -1.95E-02 | 9.23E-03 | 9.83E-01 | 202393_s_at | NM_005655 | KLF10 | -1.97E-02 | 2.37E-02 | 9.83E-01 | 201694_s_at | NM_001964 | EGR1 |
| Female subjects | | | | | | Female subjects | | | | | |
| 1.96E-02 | 3.90E-03 | 1.00E+00 | 219044_at | NM_018271 | THNSL2 | 2.82E-02 | 1.46E-01 | 1.00E+00 | Left brain | | |
| -1.81E-02 | 2.01E-01 | 1.00E+00 | 202688_at | NM_003810 | TNFSF10 | 1.89E-02 | 3.90E-03 | 1.00E+00 | 219044_at | NM_018271 | THNSL2 |
| 1.71E-02 | 1.92E-01 | 1.00E+00 | 201843_s_at | NM_004105 | EFEMP1 | -1.74E-02 | 2.01E-01 | 1.00E+00 | 202688_at | NM_003810 | TNFSF10 |
| 1.65E-02 | 6.05E-02 | 1.00E+00 | 207547_s_at | NM_007177 | FAM107A | 1.68E-02 | 1.92E-01 | 1.00E+00 | 201843_s_at | NM_004105 | EFEMP1 |
| -1.63E-02 | 4.18E-02 | 1.00E+00 | 205139_s_at | NM_005715 | UST | 1.60E-02 | 6.05E-02 | 1.00E+00 | 207547_s_at | NM_007177 | FAM107A |
| -1.58E-02 | 2.10E-01 | 1.00E+00 | 217317_s_at | AB002391 | HERC2P2,… | -1.59E-02 | 4.18E-02 | 1.00E+00 | 205139_s_at | NM_005715 | UST |
| 1.57E-02 | 1.28E-01 | 1.00E+00 | 221008_s_at | NM_031279 | AGXT2L1 | 1.54E-02 | 1.28E-01 | 1.00E+00 | 221008_s_at | NM_031279 | AGXT2L1 |
| -1.53E-02 | 1.32E-01 | 1.00E+00 | 204004_at | AI336206 | PAWR | -1.54E-02 | 2.10E-01 | 1.00E+00 | 217317_s_at | AB002391 | HERC2P2,… |
| -1.53E-02 | 2.14E-01 | 1.00E+00 | 203534_at | NM_014462 | LSM1 | -1.50E-02 | 1.32E-01 | 1.00E+00 | 204004_at | AI336206 | PAWR |
| 1.52E-02 | 7.01E-02 | 1.00E+00 | 212875_s_at | AP001745 | C21orf25 | -1.48E-02 | 2.14E-01 | 1.00E+00 | 203534_at | NM_014462 | LSM1 |
| 1.45E-02 | 1.63E-01 | 1.00E+00 | 205872_x_at | NM_022359 | PDE4DIP | 1.46E-02 | 7.01E-02 | 1.00E+00 | 212875_s_at | AP001745 | C21orf25 |
| -1.45E-02 | 1.60E-01 | 1.00E+00 | 202746_at | AL021786 | ITM2A | -1.42E-02 | 2.83E-01 | 1.00E+00 | 202687_s_at | U57059 | TNFSF10 |
| -1.45E-02 | 2.83E-01 | 1.00E+00 | 202687_s_at | U57059 | TNFSF10 | 1.41E-02 | 1.63E-01 | 1.00E+00 | 205872_x_at | NM_022359 | PDE4DIP |
| 1.41E-02 | 8.82E-02 | 1.00E+00 | 209074_s_at | AL050264 | FAM107A | -1.41E-02 | 1.60E-01 | 1.00E+00 | 202746_at | AL021786 | ITM2A |
| 1.40E-02 | 2.50E-01 | 1.00E+00 | 220182_at | NM_024103 | SLC25A23 | 1.38E-02 | 8.82E-02 | 1.00E+00 | 209074_s_at | AL050264 | FAM107A |
| -1.40E-02 | 1.13E-01 | 1.00E+00 | 215244_at | AI479306 | DGCR5 | 1.37E-02 | 1.20E-01 | 1.00E+00 | 218377_s_at | NM_016940 | RWDD2B |
| 1.39E-02 | 1.20E-01 | 1.00E+00 | 218377_s_at | NM_016940 | RWDD2B | -1.37E-02 | 1.54E-01 | 1.00E+00 | 211038_s_at | BC006312 | CROCCL1 |
| -1.39E-02 | 3.31E-03 | 1.00E+00 | 221011_s_at | NM_030915 | LBH | -1.36E-02 | 1.13E-01 | 1.00E+00 | 215244_at | AI479306 | DGCR5 |
| -1.38E-02 | 1.54E-01 | 1.00E+00 | 211038_s_at | BC006312 | CROCCL1 | -1.36E-02 | 3.31E-03 | 1.00E+00 | 221011_s_at | NM_030915 | LBH |
| 1.38E-02 | 1.73E-01 | 1.00E+00 | 205439_at | NM_000854 | GSTT2(B) | 1.33E-02 | 2.50E-01 | 1.00E+00 | 220182_at | NM_024103 | SLC25A23 |

Genes sorted by SVM-weight, bipolar versus control (stratified data)

| Expression data only | | | | | | Demographic + expression data | | | | | |
|---|---|---|---|---|---|---|---|---|---|---|---|
| All subjects | | | | | | All subjects | | | | | |
| SVM-weight | *p* -value | *q* -value | ID | GenBank | Symbol | SVM-weight | *p* -value | *q* -value | ID | GenBank | Symbol |
| -6.52E-02 | 7.53E-07 | 8.06E-03 | 202203_s_at | NM_001144 | AMFR | 6.89E-02 | 1.96E-04 | 8.34E-02 | Age | | |
| 5.82E-02 | 9.57E-03 | 1.09E-01 | 211751_at | BC005949 | PDE4DIP | -6.33E-02 | 7.53E-07 | 8.07E-03 | 202203_s_at | NM_001144 | AMFR |
| -5.03E-02 | 1.74E-03 | 1.09E-01 | 209189_at | BC004490 | FOS | 5.77E-02 | 9.57E-03 | 1.09E-01 | 211751_at | BC005949 | PDE4DIP |
| -4.99E-02 | 5.49E-03 | 1.09E-01 | 202688_at | NM_003810 | TNFSF10 | -4.98E-02 | 1.74E-03 | 1.09E-01 | 209189_at | BC004490 | FOS |
| 4.49E-02 | 4.49E-03 | 1.09E-01 | 214722_at | AW516297 | NOTCH2NL | -4.93E-02 | 5.49E-03 | 1.09E-01 | 202688_at | NM_003810 | TNFSF10 |
| -4.43E-02 | 3.80E-04 | 9.69E-02 | 202393_s_at | NM_005655 | KLF10 | 4.46E-02 | 4.49E-03 | 1.09E-01 | 214722_at | AW516297 | NOTCH2NL |
| -4.35E-02 | 2.25E-02 | 1.22E-01 | 205249_at | NM_000399 | EGR2 | -4.18E-02 | 3.80E-04 | 9.47E-02 | 202393_s_at | NM_005655 | KLF10 |
| -4.14E-02 | 1.19E-04 | 7.50E-02 | 213338_at | BF062629 | TMEM158 | -4.16E-02 | 2.25E-02 | 1.22E-01 | 205249_at | NM_000399 | EGR2 |
| -4.01E-02 | 2.18E-05 | 3.89E-02 | 201865_x_at | AI432196 | NR3C1 | -4.06E-02 | 1.19E-04 | 7.51E-02 | 213338_at | BF062629 | TMEM158 |
| 3.91E-02 | 1.57E-02 | 1.16E-01 | 202071_at | NM_002999 | SDC4 | -3.95E-02 | 2.18E-05 | 3.89E-02 | 201865_x_at | AI432196 | NR3C1 |
| 3.74E-02 | 1.57E-02 | 1.16E-01 | 222031_at | AW452796 | LOC389906,… | -3.84E-02 | 2.12E-02 | 1.20E-01 | 219683_at | NM_017412 | FZD3 |
| -3.73E-02 | 2.12E-02 | 1.21E-01 | 219683_at | NM_017412 | FZD3 | 3.81E-02 | 1.57E-02 | 1.16E-01 | 202071_at | NM_002999 | SDC4 |
| -3.38E-02 | 8.22E-06 | 2.93E-02 | 202478_at | NM_021643 | TRIB2 | 3.41E-02 | 1.57E-02 | 1.16E-01 | 222031_at | AW452796 | LOC389906,… |
| -3.30E-02 | 1.42E-04 | 8.02E-02 | 208893_s_at | BC005047 | DUSP6 | 3.22E-02 | 1.74E-02 | 1.16E-01 | 201010_s_at | NM_006472 | TXNIP |
| -3.28E-02 | 3.35E-03 | 1.09E-01 | 221579_s_at | AF062530 | NUDT3 | -3.17E-02 | 8.22E-06 | 2.93E-02 | 202478_at | NM_021643 | TRIB2 |
| -3.24E-02 | 1.23E-03 | 1.09E-01 | 208078_s_at | NM_030751 | SNF1LK | 3.16E-02 | 1.96E-02 | 1.19E-01 | 207078_at | NM_005466 | MED6 |
| 3.22E-02 | 1.96E-02 | 1.19E-01 | 207078_at | NM_005466 | MED6 | -3.14E-02 | 1.42E-04 | 8.02E-02 | 208893_s_at | BC005047 | DUSP6 |
| 3.21E-02 | 1.74E-02 | 1.16E-01 | 201010_s_at | NM_006472 | TXNIP | -3.12E-02 | 2.54E-02 | 1.22E-01 | 200800_s_at | NM_005345 | HSPA1A/B |
| -3.17E-02 | 2.54E-02 | 1.22E-01 | 200800_s_at | NM_005345 | HSPA1A/B | 3.08E-02 | 2.45E-03 | 1.09E-01 | 219230_at | NM_018286 | TMEM100 |
| 3.12E-02 | 2.45E-03 | 1.09E-01 | 219230_at | NM_018286 | TMEM100 | -3.06E-02 | 1.23E-03 | 1.09E-01 | 208078_s_at | NM_030751 | SNF1LK |
| Male subjects | | | | | | Male subjects | | | | | |
| -4.35E-02 | 1.19E-01 | 8.72E-01 | 220122_at | NM_024717 | MCTP1 | 4.21E-02 | 2.02E-04 | 8.72E-01 | Alcohol use | | |
| -3.20E-02 | 9.09E-03 | 8.72E-01 | 205249_at | NM_000399 | EGR2 | -4.16E-02 | 5.72E-02 | 8.72E-01 | Left brain | | |
| 3.12E-02 | 1.22E-01 | 8.72E-01 | 211751_at | BC005949 | PDE4DIP | -4.06E-02 | 1.19E-01 | 8.72E-01 | 220122_at | NM_024717 | MCTP1 |
| -3.10E-02 | 1.07E-02 | 8.72E-01 | 209189_at | BC004490 | FOS | -3.06E-02 | 1.07E-02 | 8.72E-01 | 209189_at | BC004490 | FOS |
| 2.75E-02 | 1.45E-01 | 8.72E-01 | 201061_s_at | M81635 | STOM | -3.02E-02 | 9.09E-03 | 8.72E-01 | 205249_at | NM_000399 | EGR2 |
| 2.69E-02 | 3.75E-02 | 8.72E-01 | 221008_s_at | NM_031279 | AGXT2L1 | 2.96E-02 | 1.22E-01 | 8.72E-01 | 211751_at | BC005949 | PDE4DIP |
| 2.61E-02 | 3.01E-02 | 8.72E-01 | 219230_at | NM_018286 | TMEM100 | 2.68E-02 | 3.75E-02 | 8.72E-01 | 221008_s_at | NM_031279 | AGXT2L1 |
| -2.61E-02 | 1.69E-02 | 8.72E-01 | 201694_s_at | NM_001964 | EGR1 | 2.63E-02 | 1.45E-01 | 8.72E-01 | 201061_s_at | M81635 | STOM |
| -2.52E-02 | 2.26E-02 | 8.72E-01 | 218948_at | AL136679 | QRSL1 | -2.45E-02 | 1.69E-02 | 8.72E-01 | 201694_s_at | NM_001964 | EGR1 |
| 2.50E-02 | 1.15E-01 | 8.72E-01 | 208949_s_at | BC001120 | LGALS3 | 2.43E-02 | 3.01E-02 | 8.72E-01 | 219230_at | NM_018286 | TMEM100 |
| -2.35E-02 | 1.37E-02 | 8.72E-01 | 202393_s_at | NM_005655 | KLF10 | -2.40E-02 | 2.26E-02 | 8.72E-01 | 218948_at | AL136679 | QRSL1 |
| -2.35E-02 | 1.01E-01 | 8.72E-01 | 215288_at | AI769824 | LOC650465 | 2.38E-02 | 1.15E-01 | 8.72E-01 | 208949_s_at | BC001120 | LGALS3 |
| 2.32E-02 | 1.58E-02 | 8.72E-01 | 205856_at | NM_015865 | SLC14A1 | 2.28E-02 | 1.58E-02 | 8.72E-01 | 205856_at | NM_015865 | SLC14A1 |
| -2.31E-02 | 4.37E-03 | 8.72E-01 | 210090_at | AF193421 | ARC | -2.25E-02 | 1.37E-02 | 8.72E-01 | 202393_s_at | NM_005655 | KLF10 |
| -2.27E-02 | 1.80E-03 | 8.72E-01 | 208891_at | BC003143 | DUSP6 | 2.22E-02 | 1.78E-02 | 8.72E-01 | 216012_at | U43604 | Unid. mRNA |
| -2.25E-02 | 3.10E-02 | 8.72E-01 | 209959_at | U12767 | NR4A3 | -2.21E-02 | 3.10E-02 | 8.72E-01 | 209959_at | U12767 | NR4A3 |
| 2.21E-02 | 1.78E-02 | 8.72E-01 | 216012_at | U43604 | Unid. mRNA | -2.21E-02 | 1.01E-01 | 8.72E-01 | 215288_at | AI769824 | LOC650465 |
| -2.20E-02 | 4.70E-02 | 8.72E-01 | 202340_x_at | NM_002135 | NR4A1 | -2.19E-02 | 4.37E-03 | 8.72E-01 | 210090_at | AF193421 | ARC |
| 2.02E-02 | 7.05E-02 | 8.72E-01 | 209291_at | AW157094 | ID4 | -2.12E-02 | 1.80E-03 | 8.72E-01 | 208891_at | BC003143 | DUSP6 |
| -2.01E-02 | 7.90E-02 | 8.72E-01 | 213338_at | BF062629 | TMEM158 | -2.09E-02 | 4.70E-02 | 8.72E-01 | 202340_x_at | NM_002135 | NR4A1 |
| Female subjects | | | | | | Female subjects | | | | | |
| -3.98E-02 | 5.50E-02 | 3.18E-01 | 202688_at | NM_003810 | TNFSF10 | 5.66E-02 | 1.72E-06 | 2.04E-02 | Age | | |
| -3.51E-02 | 9.97E-03 | 3.18E-01 | 202203_s_at | NM_001144 | AMFR | -3.88E-02 | 5.50E-02 | 3.18E-01 | 202688_at | NM_003810 | TNFSF10 |
| -3.23E-02 | 2.82E-02 | 3.18E-01 | 201222_s_at | AL527365 | RAD23B | -3.21E-02 | 9.97E-03 | 3.18E-01 | 202203_s_at | NM_001144 | AMFR |
| 3.03E-02 | 3.82E-02 | 3.18E-01 | 219525_at | NM_018242 | SLC47A1 | -3.01E-02 | 2.82E-02 | 3.18E-01 | 201222_s_at | AL527365 | RAD23B |
| 2.48E-02 | 4.89E-02 | 3.18E-01 | 211751_at | BC005949 | PDE4DIP | 2.85E-02 | 3.82E-02 | 3.18E-01 | 219525_at | NM_018242 | SLC47A1 |
| -2.32E-02 | 1.83E-03 | 3.18E-01 | 213038_at | AL031602 | RNF19B | 2.40E-02 | 4.89E-02 | 3.18E-01 | 211751_at | BC005949 | PDE4DIP |
| -2.31E-02 | 7.94E-03 | 3.18E-01 | 210317_s_at | U28936 | YWHAE | -2.20E-02 | 7.94E-03 | 3.18E-01 | 210317_s_at | U28936 | YWHAE |
| -2.30E-02 | 3.43E-05 | 3.18E-01 | 201865_x_at | AI432196 | NR3C1 | -2.19E-02 | 3.43E-05 | 2.04E-01 | 201865_x_at | AI432196 | NR3C1 |
| -2.26E-02 | 1.11E-02 | 3.18E-01 | 221579_s_at | AF062530 | NUDT3 | -2.17E-02 | 1.83E-03 | 3.18E-01 | 213038_at | AL031602 | RNF19B |
| 2.22E-02 | 3.93E-02 | 3.18E-01 | 219205_at | NM_021947 | SRR | 2.10E-02 | 3.93E-02 | 3.18E-01 | 219205_at | NM_021947 | SRR |
| -2.17E-02 | 2.99E-02 | 3.18E-01 | 209806_at | BC000893 | HIST1H2BK | -2.08E-02 | 1.11E-02 | 3.18E-01 | 221579_s_at | AF062530 | NUDT3 |
| -2.14E-02 | 1.97E-04 | 3.18E-01 | 213338_at | BF062629 | TMEM158 | -2.03E-02 | 2.99E-02 | 3.18E-01 | 209806_at | BC000893 | HIST1H2BK |
| -2.06E-02 | 9.16E-04 | 3.18E-01 | 209120_at | AL037401 | NR2F2 | -2.02E-02 | 1.97E-04 | 3.18E-01 | 213338_at | BF062629 | TMEM158 |
| 2.04E-02 | 3.50E-02 | 3.18E-01 | 210008_s_at | AA513737 | MRPS12 | -2.01E-02 | 9.16E-04 | 3.18E-01 | 209120_at | AL037401 | NR2F2 |
| 1.91E-02 | 8.55E-03 | 3.18E-01 | 207285_x_at | NM_001318 | CSHL1 | 1.92E-02 | 3.50E-02 | 3.18E-01 | 210008_s_at | AA513737 | MRPS12 |
| -1.91E-02 | 6.94E-04 | 3.18E-01 | 34031_i_at | U90269 | KRIT1 | 1.85E-02 | 8.55E-03 | 3.18E-01 | 207285_x_at | NM_001318 | CSHL1 |
| -1.89E-02 | 1.67E-02 | 3.18E-01 | 208078_s_at | NM_030751 | SNF1LK | -1.85E-02 | 6.94E-04 | 3.18E-01 | 34031_i_at | U90269 | KRIT1 |
| -1.89E-02 | 1.39E-02 | 3.18E-01 | 200800_s_at | NM_005345 | HSPA1A/B | -1.83E-02 | 2.15E-03 | 3.18E-01 | 202437_s_at | NM_000104 | CYP1B1 |
| -1.87E-02 | 5.34E-04 | 3.18E-01 | 202478_at | NM_021643 | TRIB2 | -1.80E-02 | 1.39E-02 | 3.18E-01 | 200800_s_at | NM_005345 | HSPA1A/B |
| -1.86E-02 | 2.15E-03 | 3.18E-01 | 202437_s_at | NM_000104 | CYP1B1 | 1.79E-02 | 4.30E-02 | 3.18E-01 | 219976_at | NM_015888 | HOOK1 |
